# Bioinformatics and systems-biology analysis to determine the effects of Coronavirus disease 2019 on patients with allergic asthma

**DOI:** 10.3389/fimmu.2022.988479

**Published:** 2022-09-23

**Authors:** Hongwei Fang, Zhun Sun, Zhouyi Chen, Anning Chen, Donglin Sun, Yan Kong, Hao Fang, Guojun Qian

**Affiliations:** ^1^ Affiliated Cancer Hospital and Institute of Guangzhou Medical University, Guangzhou, China; ^2^ Department of Anesthesiology, Zhongshan Hospital, Fudan University, Shanghai, China; ^3^ Department of Anesthesiology (High-Tech Branch), The First Affiliated Hospital of Anhui Medical University, Hefei, China; ^4^ Department of Anesthesiology, Minhang Hospital, Fudan University, Shanghai, China

**Keywords:** coronavirus disease 2019, allergic asthma, disease biomarker, hub gene, gene ontology, drug, systems-biology, bioinformatics

## Abstract

**Background:**

The coronavirus disease (COVID-19) pandemic has posed a significant challenge for global health systems. Increasing evidence shows that asthma phenotypes and comorbidities are major risk factors for COVID-19 symptom severity. However, the molecular mechanisms underlying the association between COVID-19 and asthma are poorly understood. Therefore, we conducted bioinformatics and systems biology analysis to identify common pathways and molecular biomarkers in patients with COVID-19 and asthma, as well as potential molecular mechanisms and candidate drugs for treating patients with both COVID-19 and asthma.

**Methods:**

Two sets of differentially expressed genes (DEGs) from the GSE171110 and GSE143192 datasets were intersected to identify common hub genes, shared pathways, and candidate drugs. In addition, murine models were utilized to explore the expression levels and associations of the hub genes in asthma and lung inflammation/injury.

**Results:**

We discovered 157 common DEGs between the asthma and COVID-19 datasets. A protein–protein-interaction network was built using various combinatorial statistical approaches and bioinformatics tools, which revealed several hub genes and critical modules. Six of the hub genes were markedly elevated in murine asthmatic lungs and were positively associated with IL-5, IL-13 and MUC5AC, which are the key mediators of allergic asthma. Gene Ontology and pathway analysis revealed common associations between asthma and COVID-19 progression. Finally, we identified transcription factor–gene interactions, DEG–microRNA coregulatory networks, and potential drug and chemical-compound interactions using the hub genes.

**Conclusion:**

We identified the top 15 hub genes that can be used as novel biomarkers of COVID-19 and asthma and discovered several promising candidate drugs that might be helpful for treating patients with COVID-19 and asthma.

## Introduction

Coronavirus disease 2019 (COVID-19) is a respiratory disease caused by severe acute respiratory syndrome coronavirus 2 (SARS-CoV-2) (1, 2). SARS-CoV-2 spreads quickly through respiratory droplets and aerosols produced by coughing and sneezing (3, 4). Coughs, wheezing, shortness of breath, and chest tightness are symptoms of asthma, which is a chronic inflammatory disease of the airways (5, 6). Both COVID-19 and asthma remain major global challenges for public health (7–9). Given the COVID-19 pandemic and the prevalence of allergic asthma, concerns have been raised regarding the possible increased susceptibility of patients with asthma to SARS-CoV-2 infection and disease severity (9, 10).

Previous studies have demonstrated a probable link between SARS-CoV-2 infection and the development of allergic asthma (11–14). Choi et al. discovered a higher prevalence of asthma in the COVID-19 patient population (2.9% versus 1.6–2.2%) (15). Conversely, Izquierdo et al. found a higher prevalence of COVID-19 amongst asthma patients than in the general population (1.41% versus 0.86%) (10). Furthermore, patients with asthma receiving biologic therapy had an even higher incidence of COVID-19 (10). Thus, both investigations imply that patients with asthma are more susceptible to COVID-19 infection than the general population, particularly those with severe asthma who are receiving biologic therapy.

Several studies have reported the potential mechanism associated with COVID-19 and asthma. SARS-CoV-2 enters host cells by binding to angiotensin-converting enzyme 2 (ACE2) *via* its spike protein, and this process is promoted by the host transmembrane protease serine 2 (TMPRSS2) (16–18). It has been proposed that allergic asthma can have a protective impact *via* inhibition of the ACE2 receptor or, more speculatively, by counterbalancing the heightened antiviral immune response seen in patients with severe COVID-19 (19, 20). Furthermore, pre-existing eosinophilia—a biomarker of allergic asthma—was found to be protective against hospitalization for COVID-19 infection in a single-center retrospective investigation (21). However, there has also been concern that higher expression of TMPRSS2 found among asthmatics may facilitate SARS-CoV-2 cell entry (22, 23).

Taken together, these findings suggest the strong association between COVID-19 and asthma. However, no definitive conclusions can be drawn yet as many factors can influence the reported incidences of (severe) COVID-19 and asthma. Therefore, it is critical to learn how COVID-19 affects patients with asthma and identify potential beneficial medications for COVID-19-positive asthmatic patients that could minimize the risk of hospitalization or death.

In this study, we discovered several critical cellular-signaling pathways and gene networks that are commonly associated with COVID-19 and asthma. Furthermore, we identified 15 hub genes that might be used as novel biomarkers for targeted therapy in patients with COVID-19 and asthma, and validated them in murine models of asthma and lung inflammation/injury. We also screened out several promising candidate drugs that might be effective therapeutic agents in this population. Herein, we analyzed potential molecular mechanisms and identified several biomarkers and drugs that might be useful for treating patients with COVID-19 and allergic asthma, using a bioinformatics and a systems-biology approach.

## Materials and methods

### Gene-expression datasets

To identify common genetic interrelations and correlations between patients with COVID-19 and allergic asthma, we used both RNA-seq and microarray analysis datasets from the Gene Expression Omnibus (GEO) database (National Center for Biotechnology Information; https://www.ncbi.nlm.nih.gov/geo/) (24). For the COVID-19 dataset, we used GEO accession number GSE171110 (25), which contains whole-blood RNA-sequencing (RNA-seq) data from patients with COVID-19 and healthy donors. The data were obtained through high throughput sequencing using the Illumina HiSeq 2500 V4 system. The allergic asthma dataset (GSE143192) (26) was collected from peripheral blood mononuclear cell samples from asthmatic patients and healthy volunteers. The sequencing data were obtained using the Agilent-078298 human ceRNA array, V1.0 4X180K. A summary of the information contained in both datasets is provided in **Table 1**.

**Table 1:** Overview of the datasets with their geo-features and quantitative measurements in this analysis.

Disease name	Geo accession	GEO platform	Total DEGs count	Up-regulated DEGs count	Down-regulated DEGs count
COVID-19	GSE171110	GPL16791	4082	2815	1267
Asthma	GSE143192	GPL22120	1137	767	370

### Identification of differentially expressed genes

A gene is considered differentially expressed when a statistically significant difference is observed under different experimental conditions at the transcriptional level (27). The key goal of this analysis was to identify DEGs between case and control data from the datasets GSE171110 and GSE143192, respectively. The DEGs were identified from the long-expression values using the “limma” package of R software (version 4.2.0) with the Benjamini–Hochberg correction to control for the false-discovery rate. Significant DEGs were detected using cut-off criteria (adjusted *P*-value or *P*-value < 0.05 and |log fold-change| ≥ 1.0). Common DEGs between both datasets were obtained using Jvenn, an online Venn-analysis tool (http://jvenn.toulouse.inra.fr/app/example.html) (28).

### Gene ontology and pathway-enrichment analysis

We used EnrichR (https://maayanlab.cloud/enrichr/) (29), a comprehensive online gene-set enrichment tool, to analyze biological processes and signaling pathways associated with the common DEGs. A *P*-value < 0.05 and a Q value < 0.25 were used as standardized metrics to quantify the top functional items and pathways.

### Protein–protein-interaction network analysis

PPIs were identified using the STRING database (https://string-db.org/) (30). The resulting PPI network was processed and examined using Cytoscape (version 3.7.1). The PPI network was built using proteins encoded by the shared DEGs between COVID-19 and asthma datasets. The Markov cluster method, included in the STRING database, was used to find gene clusters. The PPI network of frequent DEGs was built using a composite score greater than 0.4. Genes showing a significant correlation in candidate modules are referred to as hub genes (31). We then used the Cytoscape plug-in, cytoHubba, to rank and examine prominent nodes in the PPI network modules and predict hub genes.

### Gene-regulatory network analysis

We discovered DEG–microRNA (miRNA)-interaction networks and DEG–transcription factor (TF)-interaction networks using NetworkAnalyst (32). DEG–miRNA-interaction networks were discovered using the TarBase (33) and miRTarBase (34) databases. The JASPAR database (35) was utilized to analyze the TF–DEG-interaction networks. We used common DEGs for the GRN analysis to uncover the transcriptional elements and miRNAs that regulate DEGs at the post-transcriptional level.

### Evaluation of candidate drugs

We used the DSigDB database to predict protein–drug interactions and identify candidate pharmacological compounds associated with DEGs. We identified drugs targeting common DEGs between COVID-19 and asthma datasets using the Enrichr web server and the DSigDB database based on a statistical threshold of an adjusted *P*-value <0.05.

### Gene–disease-association analysis

DisGeNET (http://www.disgenet.org/) is a knowledge-management platform to integrate and standardize data for disease-associated genes and variations from diverse sources. DisGeNET currently has information related to over 24,000 diseases and characteristics, 17,000 genes, and 117,000 genomic variations (36). The platform is focused on increasing the understanding of human genetic disorders. We also used NetworkAnalyst to investigate gene–disease associations to uncover diseases and chronic issues linked to common DEGs (37).

### Mouse models of asthma and acute lung inflammation/injury

To establish the house dust mite (HDM)-induced murine asthma model (38), mice were sensitized with 5 µg of HDM (Greer Laboratories) intranasally on days 0, 1, and 2, and subsequently challenged with 5 µg of HDM intranasally on days 8 to 12 to induce allergic asthma. Control mice received PBS during both the sensitization and challenge phases. The mice were euthanized for analysis 2 days after the last challenge.

To establish the lipopolysaccharide (LPS)-induced acute lung inflammation/injury model (39), mice were anesthetized and instilled with 10 μg LPS O111 (Sigma) intranasally or PBS (control). On day 4 after intranasal LPS administration, mice were euthanized for analysis.

RNA from lungs was extracted using an RNA isolation kit (Tiangen) and reversely transcribed (Tiangen). Actin served as the internal control, and the ΔΔCt quantification method was employed to calculate the relative gene expression.

All animal procedures were approved by the Committee of Animal Experiments in Guangzhou Medical University (Project number: 2019-273). All protocols adhered to the Guide for the Care and Use of Laboratory Animals.

### Statistical analysis

Data are presented as the mean ± standard error of the mean (SEM). If the data were normally distributed, a two-tailed unpaired Student’s t-test was used to assess the differences between the two groups. A Mann-Whitney U unpaired test was used for populations that were not normally distributed. Correlation analysis was conducted with GraphPad Prism (version 8.0.1) with the Pearson method. R software (version 4.2.0) and GraphPad Prism were used for all statistical analyses. A *P*-value less than 0.05 was considered statistically significant (*, *P* < 0.05; **, *P* < 0.01; ***, *P* < 0.001; ****, *P* < 0.0001, n.s., not significant.).

## Results

### Identification of genetic relationships between asthma and COVID-19

**Figure 1:**
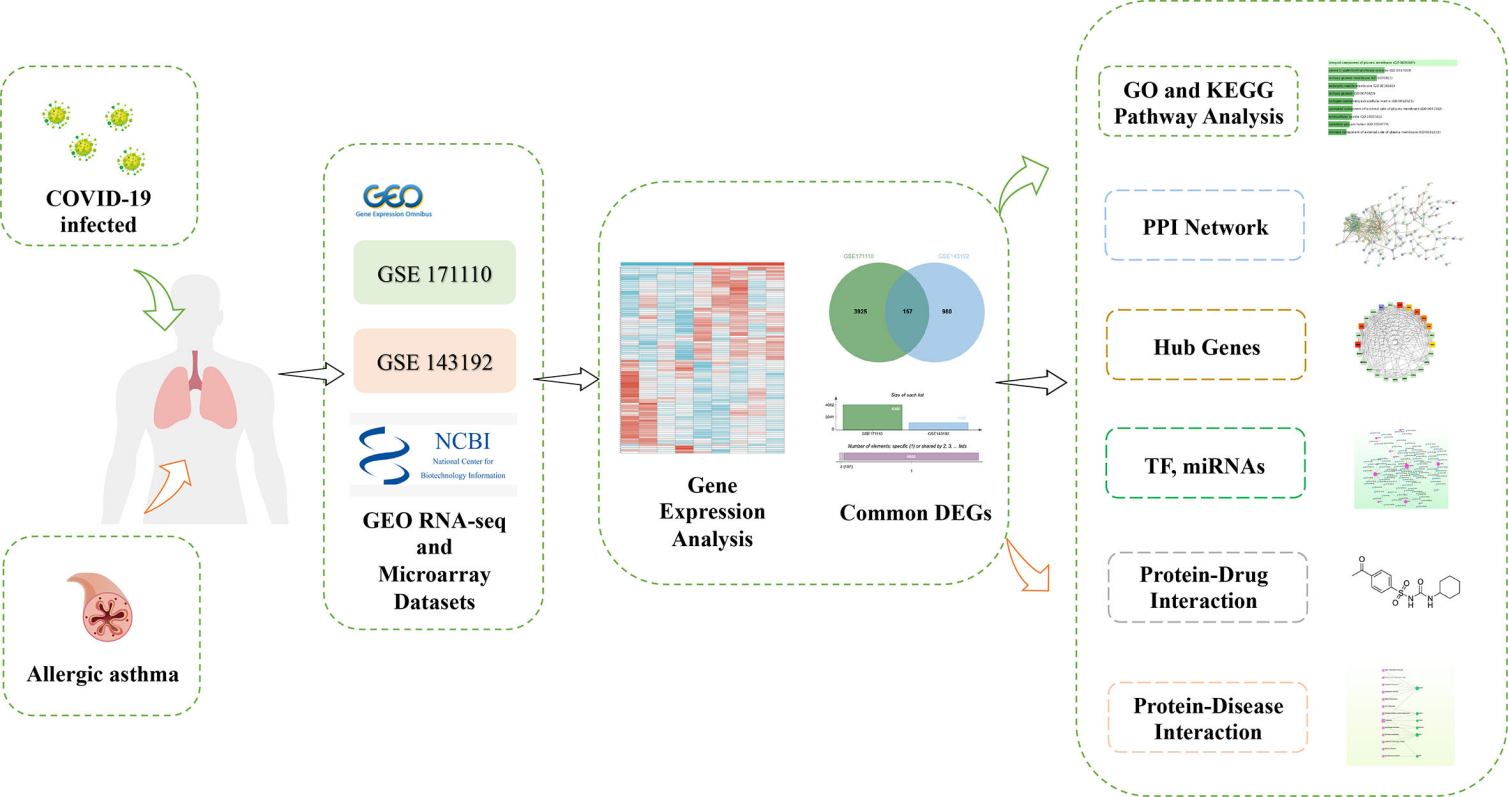
Schematic illustration of the overall general workflow of this study.

**Figure 2:**
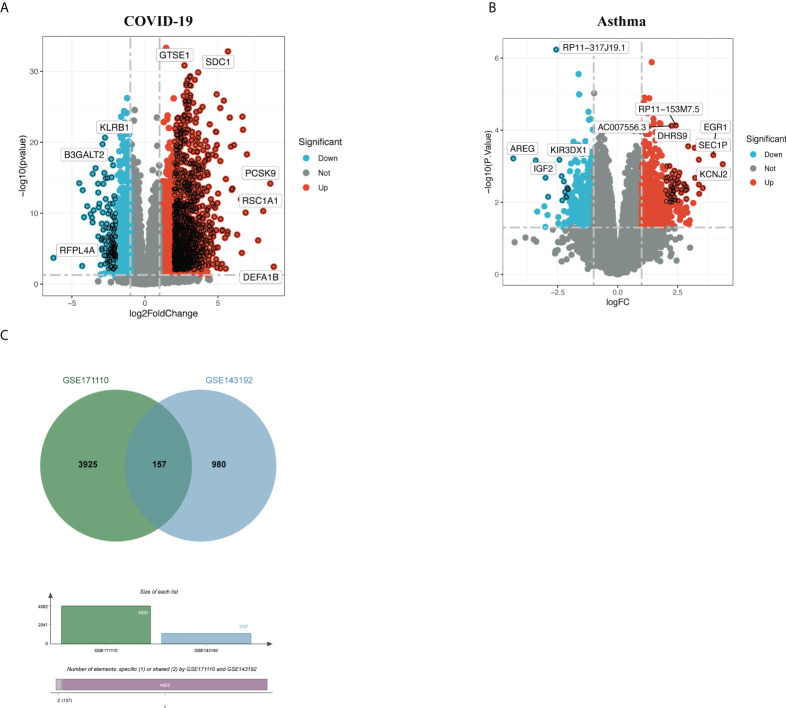
Volcano plots of **(A)** COVID-19 and **(B)** allergic asthma datasets, with genes having a log fold-change of at least 1 and *P-value* < 0.05. **(C)** The Venn diagram depicts the shared DEGs among COVID-19 and asthma.

**Figure 1** depicts the crucial procedures used in this study. To examine shared genetic interrelations between allergic asthma and COVID-19, we evaluated human RNA-seq and microarray datasets from GEO and identified the common DEGs that trigger COVID-19 and allergic asthma. First, we analyzed the transcriptomic datasets of patients with COVID-19 and identified 4,082 genes that showed differential expression compared to the corresponding levels in healthy controls. Similarly, we identified 1,137 DEGs in the asthma dataset (**Table 1**). The volcano plots presented in **Figures 2A**, **2B** show the DEGs (upregulated genes, red dots; downregulated genes, blue dots) for COVID-19 and asthma. Next, using Jvenn, a dependable web service for Venn analysis, we discovered 157 common DEGs between the asthma and COVID-19 datasets (**Figure 2C**). All 157 DEGs are listed in **Table S1**.

### Functional-enrichment analysis identifies significant cell-signaling pathways and GO terms

**Figure 3:**
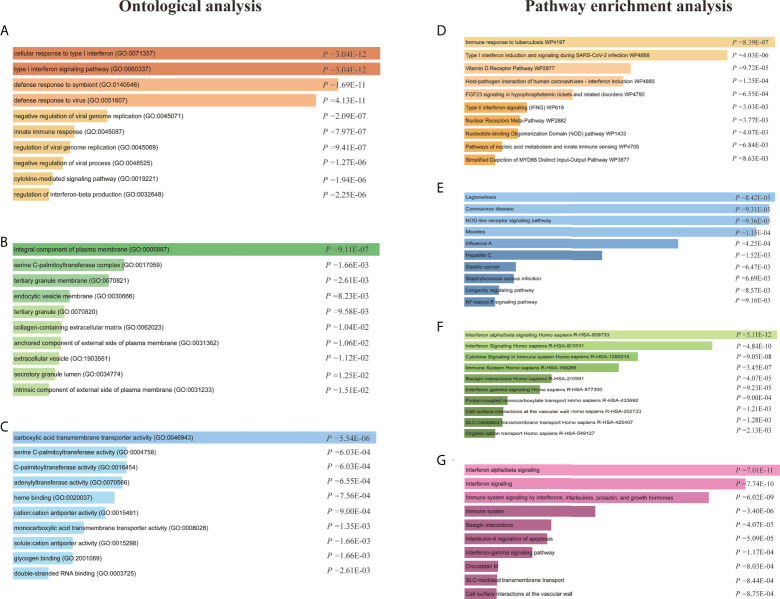
Ontological analysis and pathway enrichment analysis of shared differentially expressed genes (DEGs) between patients with COVID-19 and asthma. Ontological analysis: **(A)** biological processes, **(B)** cellular components, and **(C)** molecular function. Pathway enrichment analysis: **(D)** Wikipathway 2021, **(E)** KEGG 2021 human pathway, **(F)** Reactome pathway, and the **(G)** Bioplanet pathway.

The GO enrichment method is often used to reflect connections between genes and GO terms, whereas the Kyoto Encyclopedia of Genes and Genomes (KEGG) enrichment method can uncover gene–pathway correlations (40, 41). We used the Enrichr tool (29) to run a functional-enrichment test on common DEGs to find significantly enriched functional GO terms and cell-signaling pathways. The GO database was used as an annotation source for the GO analysis, which is divided into three categories (biological processes, cellular components, and molecular functions). **Table 2** summarizes the top 10 terms related to biological processes, molecular functions, and cellular components. The overall ontological analysis is depicted in **Figures 3A–C** as a linear bar graph for each category. The differentially expressed genes were significantly enriched in cellular response to type I interferon, integral component of plasma membrane, and carboxylic acid transmembrane transporter activity in the biological processes, cell compartments, and molecular functions subsets, respectively.

**Table 2:** Ontological analysis of common DEGs between COVID-19 and asthma.

Category	Term	P-value	Genes
GO Biological Process	cellular response to type I interferon (GO:0071357)	3.04E-12	RSAD2/IFI27/OAS1/OAS2/OAS3/MX1/IFI6/IFI35/IFIT1/IFIT3/IFIT2
	type I interferon signaling pathway (GO:0060337)	3.04E-12	RSAD2/IFI27/OAS1/OAS2/OAS3/MX1/IFI6/IFI35/IFIT1/IFIT3/IFIT2
	defense response to symbiont (GO:0140546)	1.69E-11	RSAD2/MX1/IFI6/IFIT1/RNASE2/IFIT3/IFIT2/IFIH1/IFNL1/IFI27/OAS1/OAS2/OAS3
	defense response to virus (GO:0051607)	4.13E-11	RSAD2/MX1/IFI6/IFIT1/RNASE2/IFIT3/IFIT2/IFIH1/IFNL1/IFI27/OAS1/OAS2/OAS3
	negative regulation of viral genome replication (GO:0045071)	2.09E-07	IFIH1/RSAD2/OAS1/OAS2/OAS3/MX1/IFIT1
	innate immune response (GO:0045087)	7.97E-07	CITED1/MX1/IFI6/DEFB1/IFIT1/RNASE2/IFIH1/IFNL1/IFI27/OAS1/ALPK1/TLR4/MSRB1
	regulation of viral genome replication (GO:0045069)	9.41E-07	IFIH1/RSAD2/OAS1/OAS2/OAS3/MX1/IFIT1
	negative regulation of viral process (GO:0048525)	1.27E-06	IFIH1/RSAD2/OAS1/OAS2/OAS3/MX1/IFIT1
	cytokine-mediated signaling pathway (GO:0019221)	1.94E-06	RSAD2/TNFSF14/HGF/MX1/IFI6/IRS2/IFI35/IFIT1/CXCL3/IFIT3/IFIT2/MT2A/IFNL1/IFI27/OAS1/OAS2/OAS3/FCGR1A
	regulation of interferon-beta production (GO:0032648)	2.25E-06	IFIH1/OAS1/OAS2/OAS3/SIRPA/TLR4
GO Cellular Component	integral component of plasma membrane (GO:0005887)	9.11E-07	SLC22A4/KCNK7/SIGLEC9/PTGDR2/AQP9/SLC1A3/SLC8A1/IFNL1/STBD1/CCRL2/HRH4/C3AR1/SIRPA/SLC16A8/FFAR2/FCGR1A/SLC16A3/TRPM6/CCR3/KCNJ2/KL/CD163/FZD5/FCRL5/KCNJ15/SLC16A14/TLR5/TLR4/CD200/TNFRSF21
	serine C-palmitoyltransferase complex (GO:0017059)	1.66E-03	SPTLC2/SPTSSB
	tertiary granule membrane (GO:0070821)	2.61E-03	CLEC12A/STBD1/SIRPA/MCEMP1
	endocytic vesicle membrane (GO:0030666)	8.23E-03	CD163/FZD5/NOSTRIN/WNT7A/FCGR1A
	tertiary granule (GO:0070820)	9.58E-03	CLEC12A/STBD1/SIRPA/MCEMP1/FOLR3
	collagen-containing extracellular matrix (GO:0062023)	1.04E-02	FBN2/GDF10/SRPX/CTSL/COL5A3/COL6A2/SERPING1/S100A8
	anchored component of external side of plasma membrane (GO:0031362)	1.06E-02	FOLR3/CD24
	extracellular vesicle (GO:1903561)	1.12E-02	OLFML3/COL6A2/DEFB1
	secretory granule lumen (GO:0034774)	1.25E-02	TOR4A/HGF/SERPING1/FOLR3/RNASE2/S100A8/S100A11
	intrinsic component of external side of plasma membrane (GO:0031233)	1.51E-02	FOLR3/CD24
GO Molecular Function	carboxylic acid transmembrane transporter activity (GO:0046943)	5.54E-06	SLC22A4/SLC7A5/AQP9/SLC16A8/SLC16A3/SLC16A14
	serine C-palmitoyltransferase activity (GO:0004758)	6.03E-04	SPTLC2/SPTSSB
	C-palmitoyltransferase activity (GO:0016454)	6.03E-04	SPTLC2/SPTSSB
	adenylyltransferase activity (GO:0070566)	6.55E-04	OAS1/OAS2/OAS3
	heme binding (GO:0020037)	7.56E-04	STEAP4/CBS/HBE1/CYP1B1/CYP2F1
	cation:cation antiporter activity (GO:0015491)	9.00E-04	SLC22A4/SLC8A1
	monocarboxylic acid transmembrane transporter activity (GO:0008028)	1.35E-03	SLC16A8/SLC16A3/SLC16A14
	solute:cation antiporter activity (GO:0015298)	1.66E-03	SLC22A4/SLC8A1
	glycogen binding (GO:2001069)	1.66E-03	PPP1R3B/STBD1
	double-stranded RNA binding (GO:0003725)	2.61E-03	IFIH1/OAS1/OAS2/OAS3

Top 10 terms of each category are listed.

KEGG pathway analysis is a modeling tool used to highlight how essential molecular or biological processes interact to reveal the reciprocal influences of different diseases (42). Four global databases, including KEGG, WikiPathways, Reactome, and Bioplanet, were used to assemble the most enriched pathways based on the common DEGs between asthma and COVID-19 datasets. KEGG pathway analysis revealed the following top 10 pathways: legionellosis, coronavirus disease, NOD-like receptor signaling pathway, measles, influenza A, hepatitis C, gastric cancer, *Staphylococcus aureus* infection, longevity regulating pathway, and NF-kappa B signaling pathway. The top pathways found in the examined datasets are listed in **Table 3**. **Figures 3D–G** shows more precise representations of the pathway-enrichment analysis with bar graphs.

**Table 3:** Pathway enrichment analysis of common DEGs between COVID-19 and asthma.

Category	Term	P-value	Genes
Wikipathway	Immune response to tuberculosis WP4197	8.39E-07	OAS1/MX1/IFI35/IFIT1/IFIT3
	Type I interferon induction and signaling during SARS-CoV-2 infection WP4868	4.03E-06	IFIH1/OAS1/OAS2/OAS3/TLR4
	Vitamin D Receptor Pathway WP2877	9.72E-05	KL/CDKN2B/STEAP4/CASP5/CBS/SLC8A1/S100A8/CD200
	Host-pathogen interaction of human coronaviruses - interferon induction WP4880	1.25E-04	IFIH1/OAS1/OAS2/OAS3
	FGF23 signaling in hypophosphatemic rickets and related disorders WP4790	6.55E-04	KL/FAM20C/ALPL
	Type II interferon signaling (IFNG) WP619	3.03E-03	OAS1/IFI6/IFIT2
	Nuclear Receptors Meta-Pathway WP2882	3.77E-03	SLC7A5/FGD4/HGF/CDC42EP3/CYP1B1/IRS2/SLC7A11/HSPA1A
	Nucleotide-binding Oligomerization Domain (NOD) pathway WP1433	4.07E-03	CASP5/CARD6/NLRC4
	Pathways of nucleic acid metabolism and innate immune sensing WP4705	6.84E-03	IFIH1/OAS1
	Simplified Depiction of MYD88 Distinct Input-Output Pathway WP3877	8.63E-03	TLR5/TLR4
KEGG	Legionellosis	8.42E-05	NLRC4/CXCL3/TLR5/TLR4/HSPA1A
	Coronavirus disease	9.31E-05	IFIH1/OAS1/OAS2/RPL27A/OAS3/MX1/C3AR1/TLR4/C2
	NOD-like receptor signaling pathway	9.36E-05	CASP5/OAS1/OAS2/OAS3/CARD6/NLRC4/CXCL3/TLR4
	Measles	1.15E-04	IFIH1/OAS1/OAS2/OAS3/MX1/TLR4/HSPA1A
	Influenza A	4.25E-04	IFIH1/RSAD2/OAS1/OAS2/OAS3/MX1/TLR4
	Hepatitis C	1.52E-03	RSAD2/OAS1/OAS2/OAS3/MX1/IFIT1
	Gastric cancer	6.47E-03	CDKN2B/FZD5/HGF/WNT7A/GADD45G
	Staphylococcus aureus infection	6.69E-03	C3AR1/DEFB1/FCGR1A/C2
	Longevity regulating pathway	8.57E-03	KL/IRS2/CREB5/HSPA1A
	NF-kappa B signaling pathway	9.16E-03	TNFSF14/CXCL3/TLR4/GADD45G
Reactome	Interferon alpha/beta signaling Homo sapiens R-HSA-909733	5.11E-12	RSAD2/OAS1/IFI27/OAS2/OAS3/MX1/IFI6/IFI35/IFIT1/IFIT3/IFIT2
	Interferon Signaling Homo sapiens R-HSA-913531	4.84E-10	RSAD2/MX1/IFI6/IFI35/IFIT1/IFIT3/IFIT2/MT2A/IFI27/OAS1/OAS2/OAS3/FCGR1A/FCGR1B
	Cytokine Signaling in Immune system Homo sapiens R-HSA-1280215	9.05E-08	KL/DUSP2/RSAD2/TNFSF14/HGF/MX1/IFI6/IRS2/IFI35/IFIT1/IFIT3/IFIT2/MT2A/IFNL1/IFI27/OAS1/OAS2/OAS3/FCGR1A/FCGR1B
	Immune System Homo sapiens R-HSA-168256	3.45E-07	LILRA6/SIGLEC9/IFI6/DEFB1/IRS2/IFI35/NLRC4/IFIT1/IFIT3/IFIT2/C2/IFIH1/MT2A/IFNL1/CTSL/C3AR1/FCGR1A/FCGR1B/KL/DUSP2/RSAD2/TNFSF14/HGF/MX1/IFI27/OAS1/OAS2/OAS3/SIGLEC1/TLR5/TLR4/CD200
	Basigin interactions Homo sapiens R-HSA-210991	4.07E-05	SLC7A5/SLC16A8/SLC7A11/SLC16A3
	Interferon gamma signaling Homo sapiens R-HSA-877300	9.23E-05	MT2A/OAS1/OAS2/OAS3/FCGR1A/FCGR1B
	Proton-coupled monocarboxylate transport Homo sapiens R-HSA-433692	9.00E-04	SLC16A8/SLC16A3
	Cell surface interactions at the vascular wall Homo sapiens R-HSA-202733	1.21E-03	SLC7A5/SIRPA/SLC16A8/SLC7A11/SLC16A3
	SLC-mediated transmembrane transport Homo sapiens R-HSA-425407	1.28E-03	SLC22A4/SLC7A5/SLC22A15/SLC1A3/SLC16A8/SLC7A11/SLC16A3/SLC8A1
	Organic cation transport Homo sapiens R-HSA-549127	2.13E-03	SLC22A4/SLC22A15
BioPlanet	Interferon alpha/beta signaling	7.01E-11	IFI27/OAS1/OAS2/OAS3/MX1/IFI6/IFI35/IFIT1/IFIT3/IFIT2
	Interferon signaling	7.74E-10	MX1/IFI6/IFI35/IFIT1/IFIT3/IFIT2/MT2A/IFI27/OAS1/OAS2/OAS3/FCGR1A/FCGR1B
	Immune system signaling by interferons, interleukins, prolactin, and growth hormones	6.02E-09	HGF/MX1/IFI6/IRS2/IFI35/IFIT1/IFIT3/IFIT2/MT2A/IFI27/OAS1/OAS2/OAS3/FCGR1A/FCGR1B
	Immune system	3.40E-06	HGF/MX1/IFI6/DEFB1/IRS2/IFI35/NLRC4/IFIT1/IFIT3/IFIT2/C2/IFIH1/MT2A/IFI27/OAS1/CTSL/OAS2/OAS3/TLR5/FCGR1A/TLR4/FCGR1B/CD200
	Basigin interactions	4.07E-05	SLC7A5/SLC16A8/SLC7A11/SLC16A3
	Interleukin-4 regulation of apoptosis	5.09E-05	SLC22A4/RSAD2/CTSL/MX1/IFI6/CYP1B1/ID3/RNASE2/TLR4/MS4A4A
	Interferon-gamma signaling pathway	1.17E-04	MT2A/OAS1/OAS2/OAS3/FCGR1A/FCGR1B
	Oncostatin M	8.03E-04	FBN2/MT2A/OAS1/CBS/HGF/ALPL/CXCL3/SLC16A3/S100A8
	SLC-mediated transmembrane transport	8.44E-04	SLC22A4/SLC7A5/SLC22A15/SLC1A3/SLC16A8/SLC7A11/SLC16A3/SLC8A1
	Cell surface interactions at the vascular wall	8.75E-04	SLC7A5/SIRPA/SLC16A8/SLC7A11/SLC16A3

Top 10 terms of each category are listed.

### PPI analysis identified functional networks

**Figure 4:**
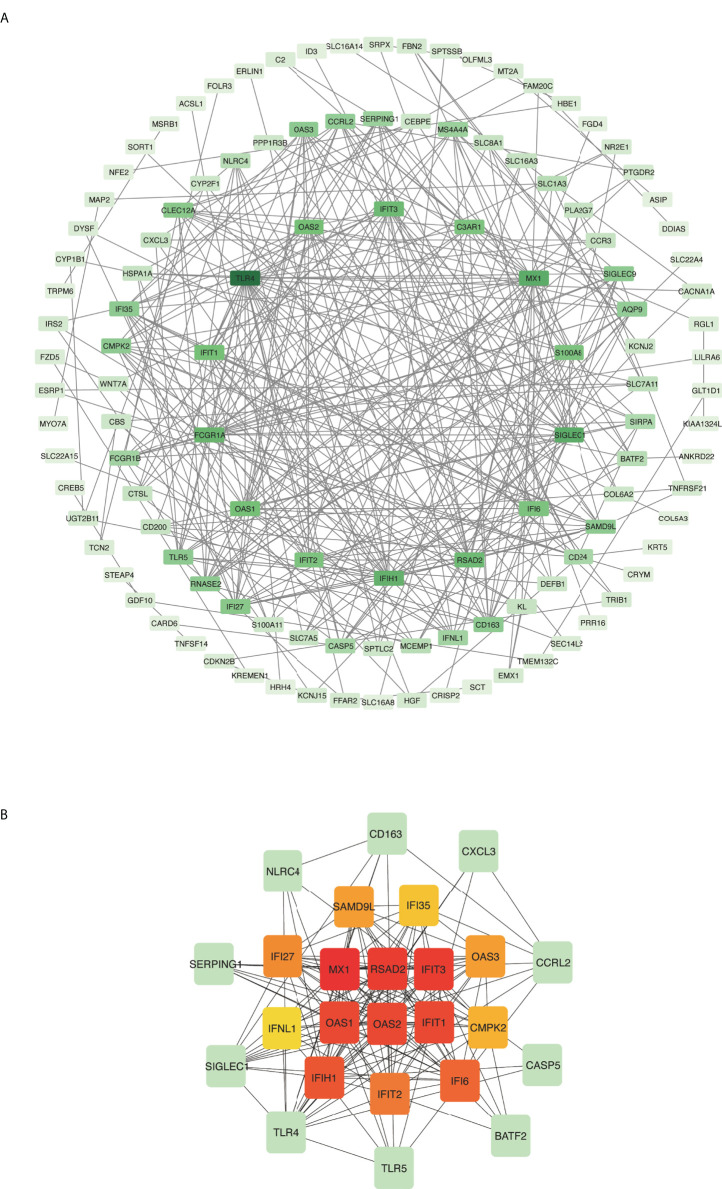
Protein–protein interaction (PPI) network and hub genes of differentially expressed genes (DEGs) common to COVID-19 and asthma. **(A)** Rectangle nodes represent DEGs and edges represent the interactions between nodes. STRING was used to create the PPI network, which was then visualized in Cytoscape. **(B)** The cytoHubba plug-in in Cytoscape was used to identify hub genes from the PPI network. The red and yellow nodes show the top 15 hub genes and their interactions with other molecules (green).

Common DEGs between COVID-19 and asthma datasets were uploaded to the STRING database to investigate PPIs and uncover common DEG interactions and adhesion pathways. **Figure 4A** shows the PPI network of common DEGs between COVID-19 and asthma datasets. The most interconnected nodes are presented as hub genes in the PPI network. CytoHubba has 11 methods for investigating networks from various viewpoints, and Maximal Clique Centrality (MCC) is the best of the 11 methods (27, 43). Based on the PPI network analysis performed using the cytoHubba plug-in of Cytoscape, we identified the top 15 (9.55%) most influential DEGs, which included *MX1*, *RSAD2*, *IFIT3*, *OAS1*, *OAS2*, *IFIT1*, *IFIH1*, *IFI6*, *IFIT2*, *IFI27*, *OAS3*, *SAMD9L*, *CMPK2*, *IFI35*, and *IFNL1* by applying the MCC method of cytoHubba. Here, the cutoff (parameter) of the topological metric for hub genes was 13 (degree). These hub genes can potentially be used as biomarkers that could lead to new therapeutic approaches for the diseases being studied. Moreover, we used the cytoHubba plug-in to establish a submodule network and adequately capture the intimate relationships and vicinity of these genes (**Figure 4B**).

### Validation of the identified hub genes using mouse models

**Figure 5:**
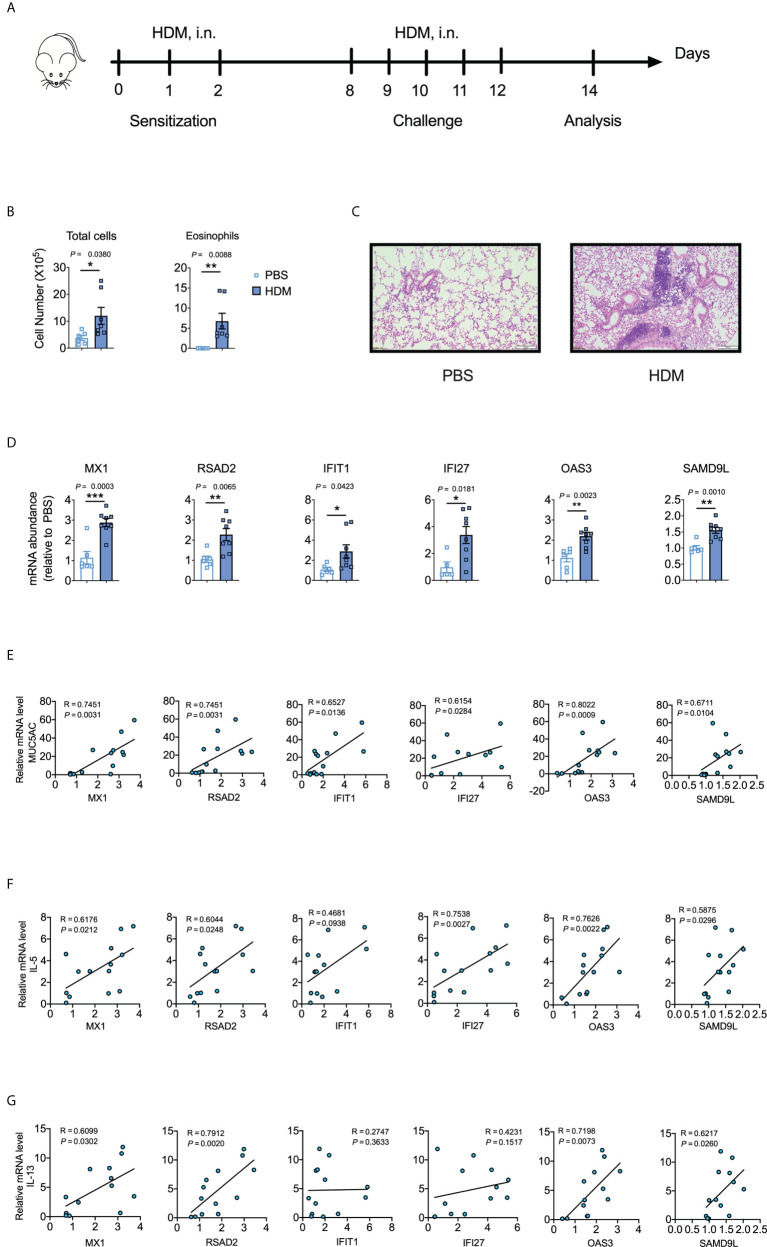
Validation of the identified hub genes with a murine model of asthma. **(A)** Schematic representation of the experimental protocol used for the murine model of house dust mite (HDM)-induced asthma. **(B)** Differential cell counts in bronchoalveolar lavage fluid (BALF) samples from PBS- or HDM-treated mice. The data shown were combined from two experiments. *n* = 6–8. **(C)** Representative hematoxylin and eosin-stained lung sections. *n* = 5–6. Scale bars, 200 μm. **(D)** Cytokine mRNA abundances in homogenized lung tissues. The data shown were combined from two experiments. *n* = 6–8. **(E-G)** Corresponding scatterplot showing the relationships between mRNA-expression levels of the identified hub genes and those of *MUC5AC*, *IL-5*, and *IL-13* in the lungs, as determined based on Spearman’s rank correlation (R). Student’s *t*-test was used to evaluate the differences. *P < 0.05, **P < 0.01, ***P < 0.001. The results shown are presented as the mean ± SEM.

To determine whether the identified hub genes could be used as biomarkers to predict asthma and COVID-induced lung inflammation/injury, we utilized murine models of HDM-induced asthma and LPS-induced lung inflammation/injury. Bronchoalveolar lavage fluid (BALF) from HDM-treated mice had more eosinophils, neutrophils, and lymphocytes than BALF from PBS-treated control mice (**Figures 5A, B**; **Figure S1A**), and the lungs from HDM-treated mice showed much more peri-bronchial and peri-vascular leukocyte infiltration (**Figure 5C**), and higher IL-4, IL-5, IL-13 and MUC5AC levels (**Figure S1B**). These results demonstrate that HDM induced allergic asthma in mice. Moreover, *MX1*, *RSAD2*, *IFIT1*, *IFI27*, *OAS3*, and *SAMD9L* were markedly elevated in HDM-treated mice (**Figure 5D**). Furthermore, *MX1*, *RSAD2*, *IFIT1*, *IFI27*, *OAS3*, and *SAMD9L* levels correlated positively with IL-5, IL-13, and MUC5AC levels (**Figures 5E–G**; **Figure S1C**), which are the key mediators of allergic asthma (5).

**Figure 6:**
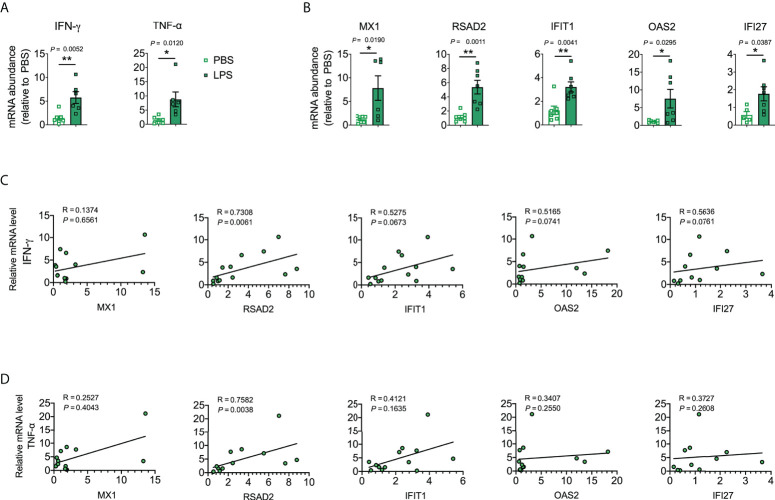
Validation of the identified hub genes with a murine lung inflammation/injury model. **(A, B)** Cytokine mRNA abundance in homogenized lung tissue. Data were combined from two experiments. *n* = 5–7. **(C, D)** Corresponding scatterplot showing the identified hub gene mRNA levels versus *IFN-γ* and *TNF-α* mRNA levels in the lung determined based on Spearman’s rank correlation (R). Student’s *t*-test was used to evaluate the differences. *, *P* < 0.05, **, *P* < 0.01. Results are shown as the mean ± SEM.

Next, we explored the lung levels of the hub genes using a murine model of LPS-induced lung inflammation/injury. We utilized an LPS-induced lung inflammation/injury model to partially mimic COVID-19-induced lung injury because we lacked a biosafety level 3 facility to develop a murine model of SARS-CoV-2 infection. *IFN-γ*, *TNF-α*, *MX1*, *RSAD2*, *IFIT1*, *OAS2*, and *IFI27* were markedly elevated in LPS-treated mice when compared to levels in the PBS control (**Figures 6A, B**). Moreover, *RSAD2* was positively correlated with *IFN-γ* and *TNF-α* (**Figures 6C, D**), which are key mediators of cytokine shock during SARS-CoV-2 infection (44).

### GRN analysis identified DEG–miRNA- and TF–gene-interaction networks

**Figure 7:**
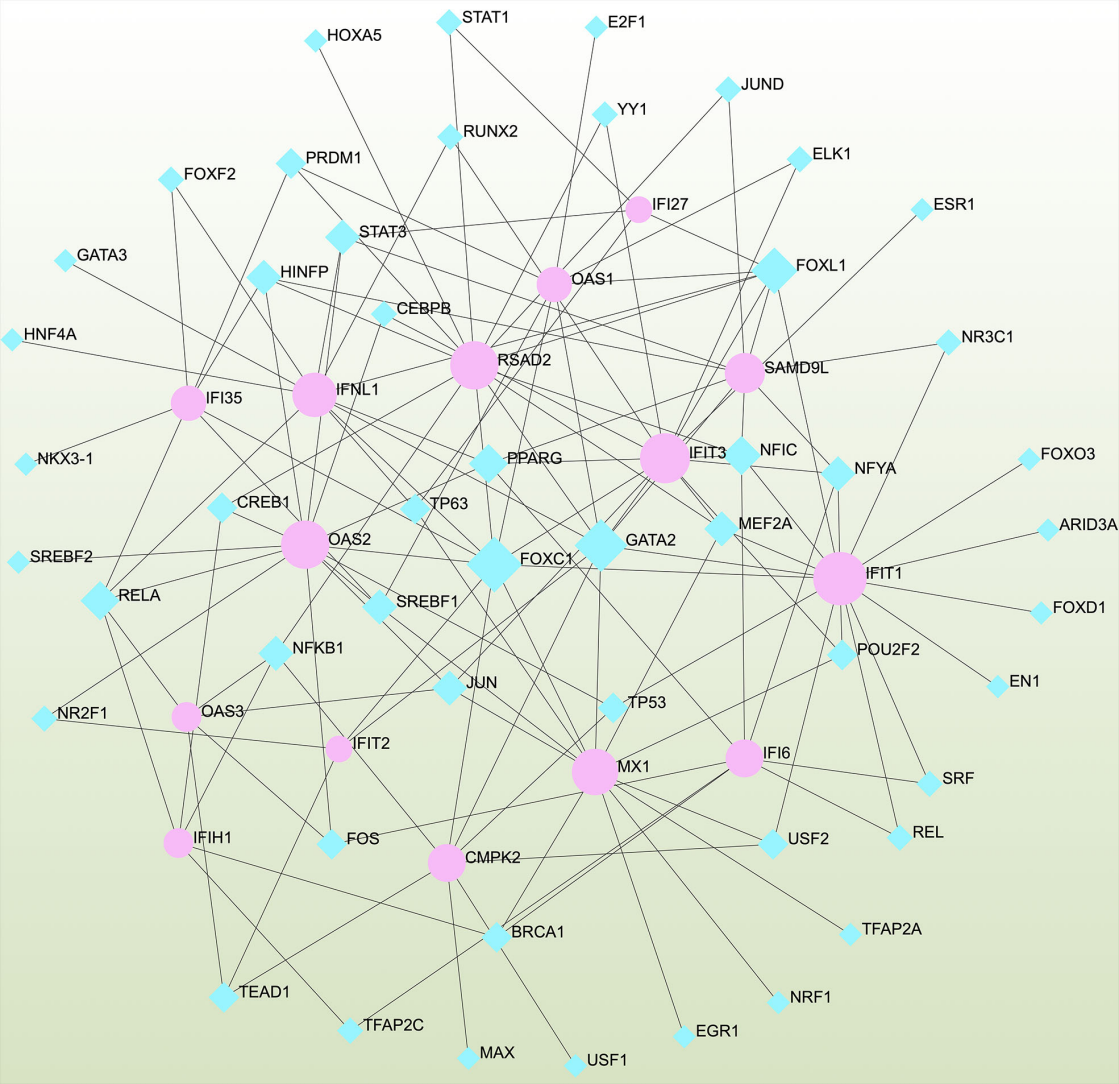
Interconnected regulatory interaction network of differentially expressed genes (DEGs)–transcription factors (TFs) created using the Network Analyst. Herein, blue nodes represent TFs, and pink nodes represent the interaction between gene symbols and TFs.

**Figure 8:**
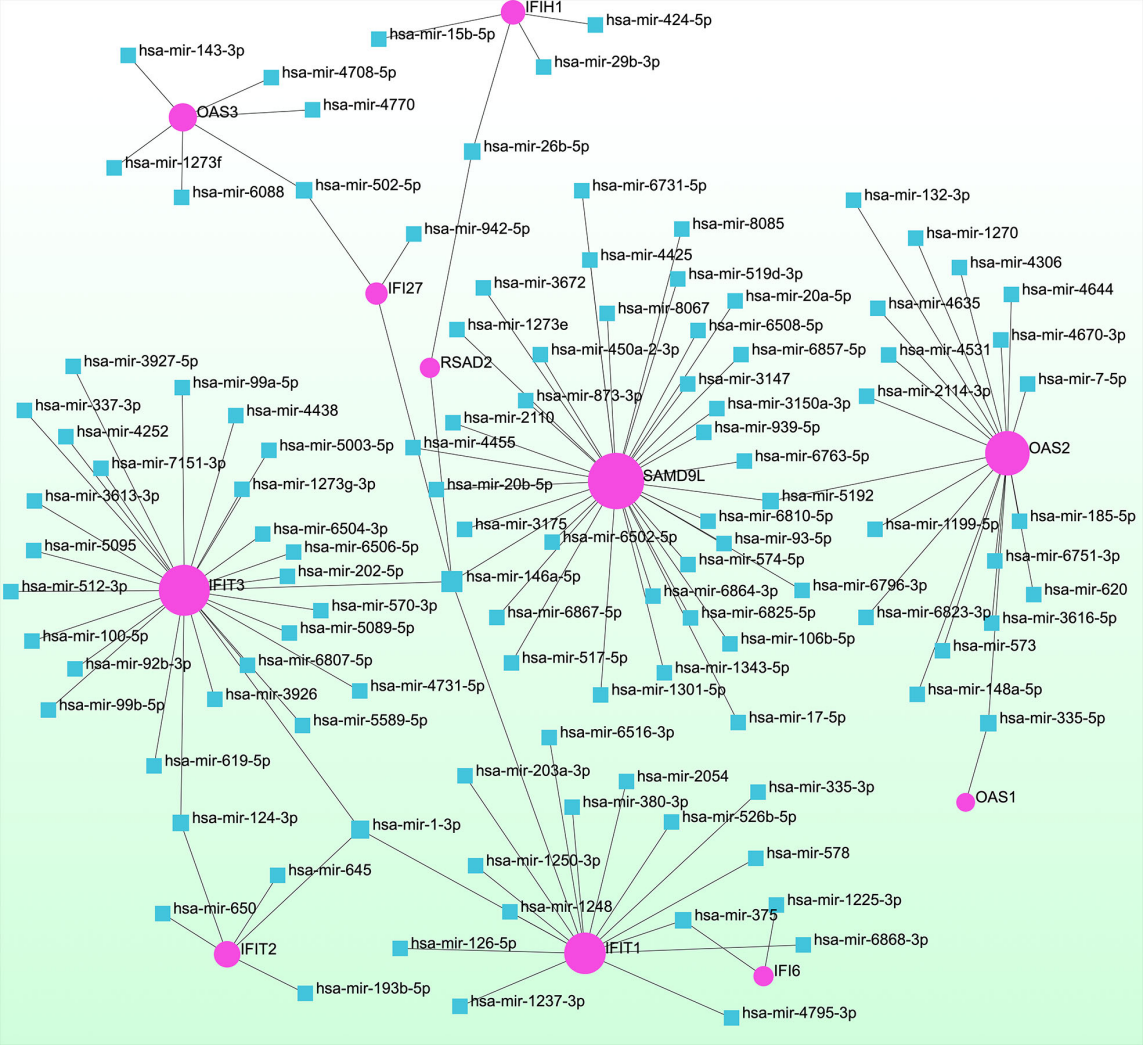
Interconnected regulatory interaction network of differentially expressed genes (DEGs)–miRNAs. Herein, the pink circle node indicates the gene symbols that interact with miRNAs.

To determine the major changes at the transcriptional level and better understand the regulatory hub proteins, we employed network analysis to discover regulatory transcription factors (TFs) and miRNAs. **Figure 7** depicts the interactions between TF regulators and the identified hub genes. The interactions of the miRNA regulators with the identified hub genes are shown in **Figure 8**. We found that 65 TFs and 119 post-transcriptional (miRNA) regulatory signals were predicted to govern multiple identified hub genes, implying a significant interplay between them. **Tables S2**, **S3** show the construction and analysis of the regulatory target TF–gene and target miRNA–gene networks, as well as the topology table, respectively.

### Identification of candidate drugs

Analyses of protein–drug interactions help to understand the structural features recommended for receptor sensitivity, which may also be useful in drug discovery (45, 46). The hub genes identified based on COVID-19–asthma interactions were used in this analysis. Ten potential therapeutic drugs were identified with Enrichr based on transcriptional characteristics from the DSigDB database, and the top 10 candidate compounds were retrieved based on their *P*-values. **Table 4** shows the top 10 enriched medications in the DSigDB database (3′-azido-3′-deoxythymidine, acetohexamide, chlorophyllin, suloctidil, estradiol, prenylamine, progesterone, benzene, clioquinol, and LY-294002).

**Table 4:** The recommended drugs for COVID-19 and asthma.

Name	*P-value*	Chemical formula	Structure
3'-Azido-3'-deoxythymidine CTD 00007047	1.82E-17	C_10_H_13_N_5_O_4_	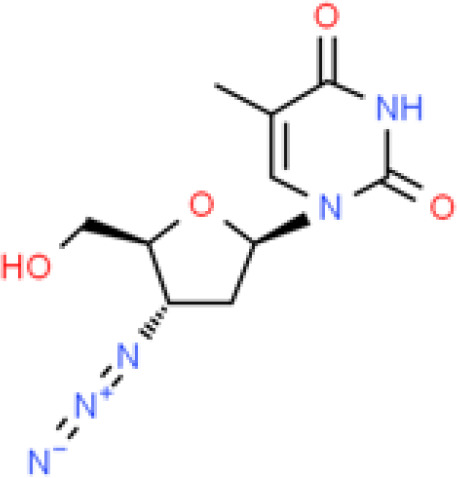
acetohexamide PC3 UP	6.68E-14	C_15_H_20_N_2_O_4_S	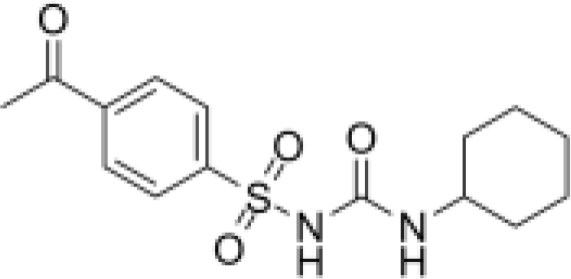
chlorophyllin CTD 00000324	1.12E-12	C_34_H_31_CuN_4_Na_3_O_6_	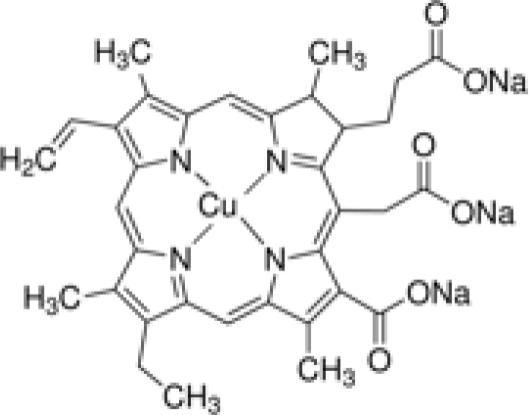
suloctidil HL60 UP	5.75E-12	C_20_H_35_NOS	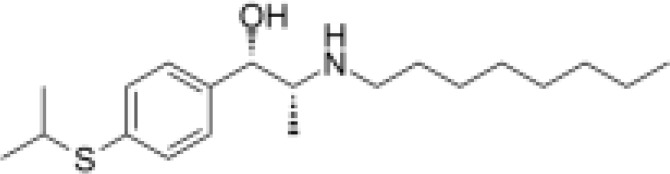
estradiol CTD 00005920	3.74E-11	C_18_H_24_O_2_	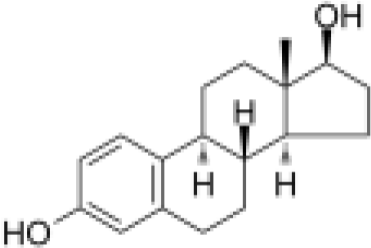
prenylamine HL60 UP	4.83E-10	C_24_H_27_N	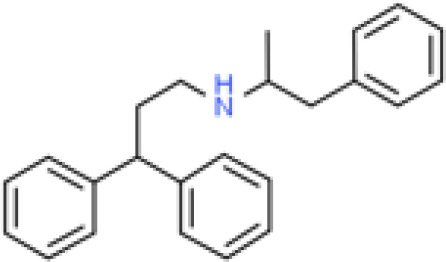
progesterone CTD 00006624	1.72E-09	C_21_H_30_O_2_	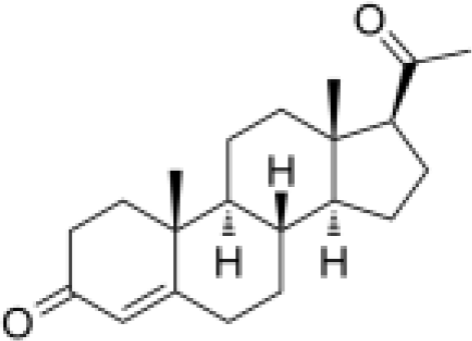
benzene CTD 00005481	2.98E-09	C_6_H_6_	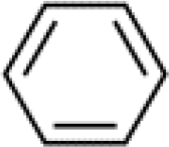
clioquinol PC3 UP	4.14E-09	C_9_H_5_ClINO	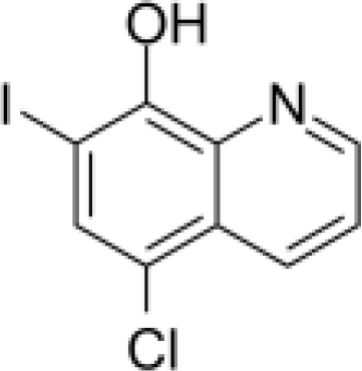
LY-294002 HL60 DOWN	7.92E-09	C_19_H_17_NO_3_	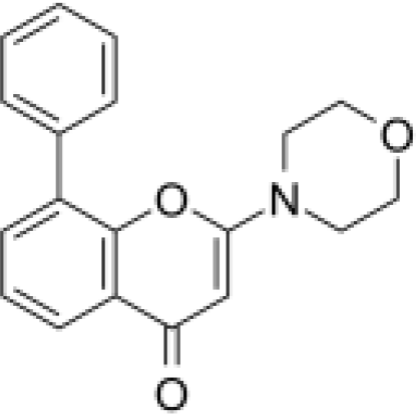

### Identification of disease associations

**Figure 9:**
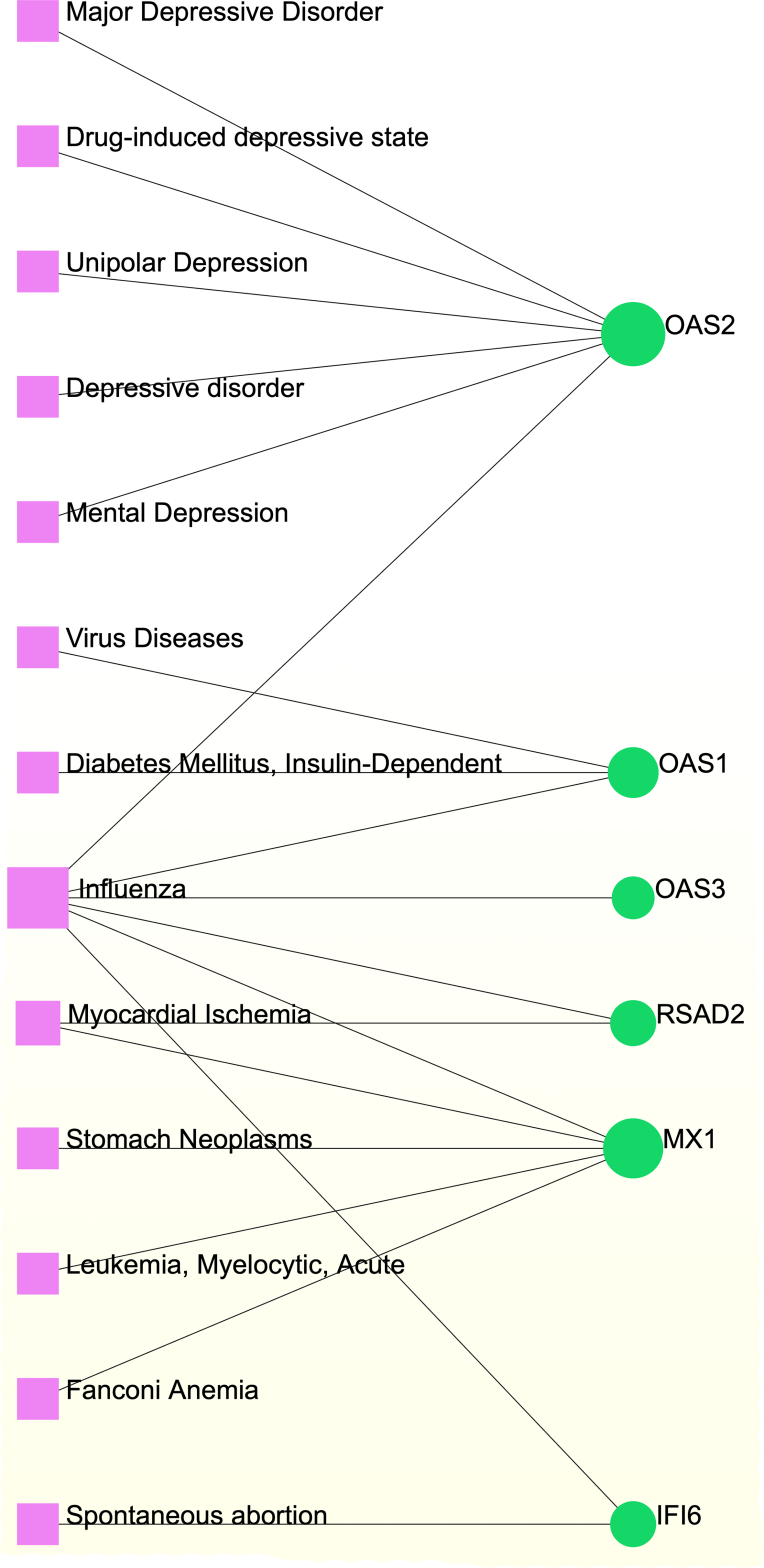
Gene-disease association network. Herein, the diseases represented by the pink square nodes and the green circle nodes indicate the gene symbols that interact with the disease.

Different diseases can be linked or correlated in certain situations, such as when they share one or more similar genes (47). Deciphering links between genes and diseases is a crucial first step in developing treatments. Our investigation of gene–disease relationships with NetworkAnalyst showed that influenza, myocardial ischemia, spontaneous abortion, Fanconi anemia, leukemia, stomach neoplasms, diabetes mellitus, viral diseases, depressive disorders, asthma, and COVID-19 were most coordinated with the hub genes identified in this study. **Figure 9** depicts the gene–disease relationships.

## Discussion

Acute asthma exacerbations are frequently caused by respiratory infections (21, 48, 49). Since the world is currently facing the COVID-19 pandemic, concerns have arisen regarding a possible increased risk of COVID-19 severity and exacerbations in people with asthma (50, 51). High-throughput sequencing datasets have become valuable resources for identifying biomarkers for many disorders (52, 53). In this context, combining bioinformatics and systems biology analytics with patient sequencing data can improve our understanding of the effects of COVID-19 on asthmatic patients and reveal new therapeutic tactics and management alternatives (54–56). In this study, network analyses were used to explore gene-expression patterns from patient datasets related to asthma and COVID-19, which yielded potential COVID-19 and asthma biomarkers and candidate drugs.

Our analysis of asthma and COVID-19 transcriptomics revealed 157 common DEGs. The biological relevance of the common DEGs was assessed through GO pathway analysis to gain insights into the pathophysiology of asthma and COVID-19. Using the Enrichr platform (29), three GO analyses were conducted with the GO database as the annotation source for biological processes (molecular activities), cellular components (genes that regulate functions), and molecular function (activities at the molecular level). For the biological processes, cellular responses to type-I interferon (IFN-I; 11 genes) and the IFN-I signaling pathway (11 genes) were among the top GO terms. IFN-I induces the production of several genes that contribute to autocrine and paracrine antiviral activities in host cells (57–59). Increasing evidence suggests that patients with severe COVID-19 have a strong IFN-I response, which contrasts with the delayed, possibly suppressed, interferon response seen during early infection (57, 60, 61). Through various pathways, a potent IFN-I response could increase hyperinflammation, leading to severe COVID-19 (57, 62, 63). Furthermore, IFN-Is inhibit type-2 asthma immunopathology and fibrotic illness by regulating ILC2 cells (64). Notably, interferon treatment benefits patients with severe asthma (64, 65).

The top GO terms based on the cellular component were “integral component of the plasma membrane” (30 genes) and the “serine C-palmitoyltransferase (SPT) complex” (two genes). During SARS-CoV-2 replication, the ribosome produces the viral membrane protein, spike glycoprotein, and envelope protein, which incorporate into the endoplasmic reticulum membrane (27, 66). The earliest and rate-limiting step in the *de novo* production of all sphingolipids is catalyzed by the human SPT complex (67, 68). SPT function is regulated by ORMDLs, with the human ORMDL3 being linked to asthma (67). The top two GO pathways in the molecular function category were “carboxylic acid transmembrane transporter activity” (six genes) and “SPT activity” (two genes). Carboxylic acid transmembrane transporter activity affects several pulmonary disorders, including asthma, acute respiratory distress syndrome, lung cancer, and pulmonary fibrosis (69–71).

KEGG pathway analysis of 157 common DEGs revealed similar pathways for asthma and COVID-19. The top KEGG pathway terms were legionellosis, coronavirus disease, NOD-like receptor signaling pathway, measles, influenza A, hepatitis C, gastric cancer, *Staphylococcus aureus* infection, longevity regulating pathway, and NF-kappa B signaling pathway. Previous evidence suggests that legionellosis, measles, and influenza A have strong relationships with acute respiratory tract infections, which are implicated in community-acquired pneumonia, acute exacerbations of chronic bronchitis, asthma, and COVID-19 (72–75).

We also established a PPI network based on DEGs to study common functional features of proteins and predict therapeutic targets. Various pathogeneses were linked to hub genes that could be important therapeutic targets or biomarkers for COVID-19 and asthma. We used the MCC method to identify the top 15 hub genes (*MX1*, *RSAD2*, *IFIT3*, *OAS1*, *OAS2*, *IFIT1*, *IFIH1*, *IFI6*, *IFIT2*, *IFI27*, *OAS3*, *SAMD9L*, *CMPK2*, *IFI35*, and *IFNL1*) associated with both asthma and COVID-19. We utilized a mouse model of allergic asthma with HDM (which can induce T-helper-cell-mediated airway inflammation reminiscent of human asthma) to ascertain whether the identified hub genes could be useful biomarkers for predicting asthma. *MX1*, *RSAD2*, *IFIT1*, *IFI27*, *OAS3*, and *SAMD9L* were markedly elevated in HDM-treated mice and positively associated with *IL-5*, *IL-13* and *MUC5AC* (key mediators of allergic asthma). Moreover, *MX1*, *RSAD2*, *IFIT1*, *OAS2*, and *IFI27* were markedly elevated in a murine model of LPS-induced lung inflammation/injury and RSAD2 was positively associated with *INF-γ* and *TNF-α*, the key mediators of cytokine shock during SARS-CoV-2 infection.

MX genes are found in nearly all vertebrate genomes and are primarily active against RNA viruses (76, 77). MX1 is a member of the effector protein family of the IFN system. MX1 induces a protective antiviral response by modulating the expression of critical molecules linked to influenza A virus lethality; these effects are amplified under IFN-ɑ stimulation (78, 79). Moreover, MX1 expression is higher in patients with COVID-19 and may have antiviral properties against SARS-COV-2 (76, 80). MX1 also has antiviral properties against other viruses, including influenza A, measles, and type-3 parainfluenza viruses (76, 79–81). In addition, flow-cytometric detection of MX1 protein expression in whole blood appears to be an easy and valuable method for studying viral infections during acute asthma exacerbations (82).

Gaskill et al. demonstrated that RASD2 plays a role in idiopathic pulmonary fibrosis (IPF), asthma, and chronic obstructive pulmonary disease (COPD) (83). Furthermore, RASD2 has been implicated as a target for SARS-CoV-2 infection (27). IFN-induced proteins with tetratricopeptide repeats (IFITs), such as IFIT1, IFIT2, and IFIT3, mediate IFN-related pathways and may suppress viral replication (84, 85), suggesting that they could be targeted to inhibit SARS-CoV-2 replication. OAS1, OAS2, and OAS3 homologous are members of the 2′-5′-oligoadenylate synthetase (OAS) family, which inhibits cellular protein synthesis and confers resistance to viral infection (86). Polymorphisms in the interferon-stimulated gene, OAS1, have been linked to host immune responses against several classes of viral infections (86, 87), and extracellular OAS1 may play a role in antiviral immune responses (88). A two-sample Mendelian randomization study of 931 proteins showed that increased OAS1 levels in the non-infectious state strongly correlated with reduced risks for SARS-CoV-2 susceptibility and COVID-19 severity (86).

We also discovered links between COVID-19 and asthma in terms of TFs and miRNAs. TFs drive mRNA expression, whereas miRNAs regulate gene expression *via* RNA silencing at the post-transcription level (89, 90). Thus, TFs and miRNAs play essential roles in disease development. Our analysis revealed relationships among the common DEGs, TFs, and miRNAs. We identified TFs, such as GATA3, STAT3, STAT1, CEBPB, FOS, FOXD1, MAX, GATA2, RELA, and JUN, which are associated with diffident types of respiratory diseases, such as asthma, COPD, IPF, and COVID-19 (88, 91–100). In the context of determining the relationship between DEGs and miRNAs, hsa-miR-335-5p, hsa-miR-146a-5p, hsa-miR-5192, hsa-miR-375, and hsa-miR-124-3p found to be associated with the pathogenesis and exacerbation of asthma (101–104). In patients with asthma treated with inhaled corticosteroids, hsa-miR-335-5p was strongly related to changes in the FEV1 ratio (102). MiR-146a is an immune-regulatory miRNA that executes key functions in allergies and asthma (105, 106). An miR-146a polymorphism has been linked to asthma in several studies (101, 105, 106). Previous data implicated miR-146a in immunoglobulin E (IgE) synthesis and promoting the IgE-class transition in B cells (101, 107). Furthermore, children with asthma had considerably greater plasma miR-146a levels than healthy controls (101, 108). In asthmatic patients, hsa-miR-124-3p was adversely linked to the risk of exacerbation, severity, and inflammation, but was positively associated with lung function (104). Remarkably, we also identified five miRNAs (hsa-miR-1-3p, hsa-miR-17-5p, hsa-miR-20a-5p, hsa-miR-5192, and hsa-miR-26b-5p) that were predicted to be associated with DEGs in patients with COVID-19 (109–111). Most of these miRNAs have been implicated in tumorigenesis of several types of cancer, particularly lung cancer (112–115).

Our gene–disease analysis predicted relationships between common DEGs and various disorders, including COVID-19. For example, several genes related to depressive disorders (major depressive disorder, drug-induced depressive state, unipolar depression, and mental depression) were found in the study. Mental disorders are major causes of global health burdens, with depressive and anxiety disorders being the leading contributors (116–118). Mental depression and major depressive disorder appear to synergistically influence asthma control and the quality of life (119–121). The COVID-19 pandemic has exacerbated many determinants of poor mental health (116, 122) by negatively influencing the mental health of those with anxiety, depression, or obsessive–compulsive disorders, necessitating strict therapeutic monitoring (116, 122, 123). Furthermore, our network analysis revealed associations with the identified DEGs and other viral diseases, such as influenza. Acute asthma exacerbations are frequently caused by respiratory viruses such as influenza (124, 125). The danger of co-infection with influenza A virus and SARS-CoV-2 is a serious concern for public health officials and clinicians (126, 127). Furthermore, our results suggest that individuals with COVID-19 and asthma can also be affected by myocardial ischemia, stomach neoplasms, acute myelocytic leukemia, Fanconi anemia, spontaneous abortion, and insulin-dependent diabetes.

Several substances and drugs have been tested as potential COVID-19 therapeutic agents. Remdesivir, a new nucleoside analog with broad-spectrum antiviral activity against RNA viruses, was the first licensed treatment for severe COVID-19 (128). Furthermore, favipiravir, a new antiviral medication that inhibits viral transcription and replication by competitively inhibiting RNA-dependent RNA polymerase, has been used to treat SARS-CoV-2 infection (129). We discovered that 3′-azido-3′-deoxythymidine (also known as azidothymidine, zidovudine, or Retrovir), acetohexamide, chlorophyllin, suloctidil, estradiol, prenylamine, progesterone, benzene, clioquinol, and LY-294002 are candidate drugs for treating COVID-19 and asthma. Interestingly, 3′-azido-3′-deoxythymidine is a nucleoside reverse-transcriptase inhibitor (NRTI) that was the first drug approved for treating acquired immunodeficiency syndrome, which is caused by human immunodeficiency virus (HIV) (130, 131). The approval of zidovudine and other HIV NRTIs by the Food and Drug Administration accelerated the development of antivirals for various viruses, including SARS-CoV-2 (132). A case study on COVID-19 revealed zidovudine as a drug candidate for SARS-CoV-2 (133). Acetohexamide is a first-generation sulfonylurea derivative used to treat type-2 diabetes, especially in people whose blood glucose cannot be controlled by diet (134, 135). Acetohexamide also shows promise for disrupting SARS-CoV-2 binding and replication by targeting cell surface-binding immunoglobins (136). Chlorophyllin is a central component generated from chlorophyll that has been widely employed as a green pigment in the food industry (137, 138). Previous findings demonstrated that chlorophyllin significantly suppresses IL-6 (138, 139). Chlorophyllin could be used to alleviate harsh immune-modulated outcomes in patients infected with SARS-CoV-2 by blocking IL-6 trans-signaling, thereby decreasing proinflammatory cytokine effects, lymphocyte recruitment, and neutrophil overproduction (137).

Progesterone and estradiol were also identified as candidate drugs. These endogenous reproductive hormones are abundantly produced in the periphery by the adrenal glands and ovaries and *de novo* by the brain (140). They play significant physiological roles by altering inflammatory processes and behaviors (141, 142). Recently, progesterone was demonstrated to mitigate the severity of COVID-19 pneumonia in a Syrian hamster model (143). Moreover, patients infected with SARS-CoV-2 have higher progesterone levels, which are linked to decreased COVID-19 severity (140).

Another candidate drug identified here was suloctidil, which is a new medication being tested in several clinical trials for its potential use against dementia and thrombotic diseases (144, 145). Moreover, prenylamine, iodochlorhydroxyquin, clioquinol, and LY294002 were also found as drug candidates in this study. The World Health Organization has classified prenylamine as a class V calcium channel antagonist that blocks diphenylalkylamine calcium channels (146, 147). Iodochlorhydroxyquin, also known as clioquinol, is a type of mycophenolic acid with antimicrobial and antifungal properties. Skin infections can be treated with topical medicines containing clioquinol (148). Furthermore, clioquinol was once widely used as an anti-infective, particularly for diarrhea (149). LY294002 is a highly selective inhibitor of phosphatidylinositol 3 kinase (150, 151). Beyond respiratory diseases, several other conditions are associated with SARS-CoV-2 infection, including thrombosis, blood-related problems, diarrhea, and different types of cancer. Therefore, those predicted drugs are also candidates for the treatment of other COVID-19-related diseases.

Despite presenting some interesting findings, this study has some limitations. These results, including hub genes, regulatory networks, and candidate drug identification, were obtained from bioinformatics calculations and analyses and the hub genes were validated using a murine HDM-induced asthma model and LPS-induced lung inflammation/injury model. However, the murine LPS-induced lung inflammation/injury model can only partially mimic COVID-19-induced lung injury, as acute lung inflammation/injury in this model is caused by opportunistic gram-negative bacteria (39, 152). Thus, further research studies and clinical trials are needed to validate the biological functions of the hub genes, as well as the efficacy and safety of the discovered candidate drugs.

In conclusion, we utilized transcriptomic analysis to identify common pathways and molecular biomarkers in asthma and COVID-19 to help understand the link between the SARS-CoV-2 infection and asthma. We identified 157 common DEGs between COVID-19 and asthma and conducted gene-expression analysis to discover the relevant GO terms and cell-signaling pathways. In addition, we constructed a PPI network based on the common DEGs and identified the top 15 hub genes from the PPI network and validated their association with asthma and COVID-19 infection utilizing murine models that mimic asthma and lung inflammation/injury. Finally, several drugs and drug–target interactions were identified to be associated with the 15 hub genes. Many vaccines are being employed to combat the COVID-19 pandemic, but SARS-CoV-2 continues to acquires novel mutations. COVID-19 can only be defeated by developing new and effective COVID-19 vaccines and therapies. We hope that the findings of this study will provide key insights that may help in developing novel drugs or repurposing existing therapeutic agents to combat COVID-19, particularly in patients who suffer from asthma.

## Data availability statement

The original contributions presented in the study are included in the article/**Supplementary Material**. Further inquiries can be directed to the corresponding authors.

## Ethics statement

This study was reviewed and approved by the Committee of Animal Experiments in Guangzhou Medical University (Project number: 2019-273).

## Author contributions

GQ and HF conceptualized the study. HWF, ZS and ZC collected the transcriptome and clinical data. HWF, ZS, ZC, AC, DS and YK conducted the experiments and participated in the data analysis. GQ and HWF drafted the manuscript. HF revised the final manuscript. All authors contributed to the article and approved the submitted version.

## Funding

This work was supported by grants from the National Natural Science Foundation of China (81901633 to GQ; 81971863 to HF), the Natural Science Foundation of Shanghai, China (22ZR1444700 to HF), and the Guangzhou Key Medical Discipline Construction Project Fund.

## Acknowledgments

We thank all the patients who participated in this study and donated samples, as well as the GEO database for providing their platform. We thank Editage for English language editing.

## Conflict of interest

The authors declare that the research was conducted in the absence of any commercial or financial relationships that could be construed as a potential conflict of interest.

## Publisher’s note

All claims expressed in this article are solely those of the authors and do not necessarily represent those of their affiliated organizations, or those of the publisher, the editors and the reviewers. Any product that may be evaluated in this article, or claim that may be made by its manufacturer, is not guaranteed or endorsed by the publisher.

## Abbreviations

BALF, bronchoalveolar lavage fluid; COVID-19, coronavirus disease 2019; DEG, differentially expressed gene; GO, Gene Ontology; GRN, gene-regulatory network; HDM, house dust mite; HIV, human immunodeficiency virus; IFN-I, type-I interferon; IgE, immunoglobulin E; IPF, idiopathic pulmonary fibrosis; KEGG, Kyoto Encyclopedia of Genes and Genomes; LPS, lipopolysaccharide; miRNA, microRNA; PPI, protein–protein interaction; SARS-CoV-2, severe acute respiratory syndrome coronavirus 2; SEM, standard error of the mean; TF, transcription factor.
