# Supplementary material for: Bioinformatics and systems-biology analysis to determine the effects of Coronavirus disease 2019 on patients with allergic asthma
Source: Front Immunol. 2022 Sep 23;13:988479. doi: 10.3389/fimmu.2022.988479 (PMC9537444; doi:10.3389/fimmu.2022.988479)
Supplement: Supplementary file 1 [file Table_1.docx]

**Table S1** | Common genes of asthma and COVID-19 (n=157).

| Symbol | Gene ID | Gene name |
| --- | --- | --- |
| ACSL1 | 2180 | acyl-CoA synthetase long chain family member 1 |
| AGAP1 | 116987 | ArfGAP with GTPase domain, ankyrin repeat and PH domain 1 |
| ALPK1 | 80216 | alpha kinase 1 |
| ALPL | 249 | alkaline phosphatase, biomineralization associated |
| ANKRD22 | 118932 | ankyrin repeat domain 22 |
| ANKRD34B | 340120 | ankyrin repeat domain 34B |
| AQP9 | 366 | aquaporin 9 |
| ASIP | 434 | agouti signaling protein |
| B3GNT8 | 374907 | UDP-GlcNAc:betaGal beta-1,3-N-acetylglucosaminyltransferase 8 |
| BATF2 | 116071 | basic leucine zipper ATF-like transcription factor 2 |
| C2 | 717 | complement C2 |
| C3AR1 | 719 | complement C3a receptor 1 |
| CACNA1A | 773 | calcium voltage-gated channel subunit alpha1 A |
| CARD6 | 84674 | caspase recruitment domain family member 6 |
| CASP5 | 838 | caspase 5 |
| CBS | 875 | cystathionine beta-synthase |
| CCR3 | 1232 | C-C motif chemokine receptor 3 |
| CCRL2 | 9034 | C-C motif chemokine receptor like 2 |
| CD163 | 9332 | CD163 molecule |
| CD200 | 4345 | CD200 molecule |
| CD24 | 100133941 | CD24 molecule |
| CDC42EP3 | 10602 | CDC42 effector protein 3 |
| CDKN2B | 1030 | cyclin dependent kinase inhibitor 2B |
| CEBPE | 1053 | CCAAT enhancer binding protein epsilon |
| CITED1 | 4435 | Cbp/p300 interacting transactivator with Glu/Asp rich carboxy-terminal domain 1 |
| CLEC12A | 160364 | C-type lectin domain family 12 member A |
| CMPK2 | 129607 | cytidine/uridine monophosphate kinase 2 |
| COL5A3 | 50509 | collagen type V alpha 3 chain |
| COL6A2 | 1292 | collagen type VI alpha 2 chain |
| CREB5 | 9586 | cAMP responsive element binding protein 5 |
| CRISP2 | 7180 | cysteine rich secretory protein 2 |
| CRYM | 1428 | crystallin mu |
| CTSL | 1514 | cathepsin L |
| CXCL3 | 2921 | C-X-C motif chemokine ligand 3 |
| CYP1B1 | 1545 | cytochrome P450 family 1 subfamily B member 1 |
| CYP2F1 | 1572 | cytochrome P450 family 2 subfamily F member 1 |
| DDIAS | 220042 | DNA damage induced apoptosis suppressor |
| DEFB1 | 1672 | defensin beta 1 |
| DHRS9 | 10170 | dehydrogenase/reductase 9 |
| DRAM1 | 55332 | DNA damage regulated autophagy modulator 1 |
| DUSP2 | 1844 | dual specificity phosphatase 2 |
| DYSF | 8291 | dysferlin |
| EMX1 | 2016 | empty spiracles homeobox 1 |
| ERLIN1 | 10613 | ER lipid raft associated 1 |
| ESRP1 | 54845 | epithelial splicing regulatory protein 1 |
| FAM20C | 56975 | FAM20C golgi associated secretory pathway kinase |
| FBN2 | 2201 | fibrillin 2 |
| FCGR1A | 2209 | Fc gamma receptor Ia |
| FCGR1BP | 2210 | Fc gamma receptor Ib, pseudogene |
| FCRL5 | 83416 | Fc receptor like 5 |
| FFAR2 | 2867 | free fatty acid receptor 2 |
| FGD4 | 121512 | FYVE, RhoGEF and PH domain containing 4 |
| FOLR3 | 2352 | folate receptor gamma |
| FZD5 | 7855 | frizzled class receptor 5 |
| GADD45G | 10912 | growth arrest and DNA damage inducible gamma |
| GDF10 | 2662 | growth differentiation factor 10 |
| GLT1D1 | 144423 | glycosyltransferase 1 domain containing 1 |
| GRTP1 | 79774 | growth hormone regulated TBC protein 1 |
| HBE1 | 3046 | hemoglobin subunit epsilon 1 |
| HGF | 3082 | hepatocyte growth factor |
| HIGD1C | 613227 | HIG1 hypoxia inducible domain family member 1C |
| HRH4 | 59340 | histamine receptor H4 |
| HSPA1A | 3303 | heat shock protein family A (Hsp70) member 1A |
| IFI6 | 2537 | interferon alpha inducible protein 6 |
| IFIT3 | 3437 | interferon induced protein with tetratricopeptide repeats 3 |
| IFNL1 | 282618 | interferon lambda 1 |
| KCNK7 | 10089 | potassium two pore domain channel subfamily K member 7 |
| KLHL30 | 377007 | kelch like family member 30 |
| KREMEN1 | 83999 | kringle containing transmembrane protein 1 |
| MANSC1 | 54682 | MANSC domain containing 1 |
| MS4A4A | 51338 | membrane spanning 4-domains A4A |
| MSRB1 | 51734 | methionine sulfoxide reductase B1 |
| NFE2 | 4778 | nuclear factor, erythroid 2 |
| OAS1 | 4938 | 2'-5'-oligoadenylate synthetase 1 |
| OAS2 | 4939 | 2'-5'-oligoadenylate synthetase 2 |
| OR52K2 | 119774 | olfactory receptor family 52 subfamily K member 2 |
| PPP1R3B | 79660 | protein phosphatase 1 regulatory subunit 3B |
| PRB3 | 5544 | proline rich protein BstNI subfamily 3 |
| RNASE2 | 6036 | ribonuclease A family member 2 |
| S100A8 | 6279 | S100 calcium binding protein A8 |
| SAMD9L | 219285 | sterile alpha motif domain containing 9 like |
| SCT | 6343 | secretin |
| SEC14L2 | 23541 | SEC14 like lipid binding 2 |
| SEC1P | 653677 | secretory blood group 1, pseudogene |
| SERINC2 | 347735 | serine incorporator 2 |
| SERPING1 | 710 | serpin family G member 1 |
| SIGLEC1 | 6614 | sialic acid binding Ig like lectin 1 |
| SIGLEC9 | 27180 | sialic acid binding Ig like lectin 9 |
| SIRPA | 140885 | signal regulatory protein alpha |
| SLC16A14 | 151473 | solute carrier family 16 member 14 |
| SLC16A3 | 9123 | solute carrier family 16 member 3 |
| SLC16A8 | 23539 | solute carrier family 16 member 8 |
| SLC1A3 | 6507 | solute carrier family 1 member 3 |
| SLC22A15 | 55356 | solute carrier family 22 member 15 |
| SLC22A4 | 6583 | solute carrier family 22 member 4 |
| SLC7A11 | 23657 | solute carrier family 7 member 11 |
| SLC7A5 | 8140 | solute carrier family 7 member 5 |
| SLC8A1 | 6546 | solute carrier family 8 member A1 |
| SMIM10 | 644538 | small integral membrane protein 10 |
| SORT1 | 6272 | sortilin 1 |
| SPDEF | 25803 | SAM pointed domain containing ETS transcription factor |
| SPTLC2 | 9517 | serine palmitoyltransferase long chain base subunit 2 |
| SPTSSB | 165679 | serine palmitoyltransferase small subunit B |
| SRPX | 8406 | sushi repeat containing protein X-linked |
| STBD1 | 8987 | starch binding domain 1 |
| STEAP4 | 79689 | STEAP4 metalloreductase |
| TBC1D2 | 55357 | TBC1 domain family member 2 |
| TCN2 | 6948 | transcobalamin 2 |
| TLR4 | 7099 | toll like receptor 4 |
| TLR5 | 7100 | toll like receptor 5 |
| TMEM132C | 92293 | transmembrane protein 132C |
| TNFRSF21 | 27242 | TNF receptor superfamily member 21 |
| TNFSF14 | 8740 | TNF superfamily member 14 |
| TOR4A | 54863 | torsin family 4 member A |
| TRIB1 | 10221 | tribbles pseudokinase 1 |
| TRPM6 | 140803 | transient receptor potential cation channel subfamily M member 6 |
| UGT2B11 | 10720 | UDP glucuronosyltransferase family 2 member B11 |
| WNT7A | 7476 | Wnt family member 7A |
| ZNF229 | 7772 | zinc finger protein 229 |
| ZNF541 | 84215 | zinc finger protein 541 |
| ZNF860 | 344787 | zinc finger protein 860 |
