# Supplementary material for: Bioinformatics and systems-biology analysis to determine the effects of Coronavirus disease 2019 on patients with allergic asthma
Source: Front Immunol. 2022 Sep 23;13:988479. doi: 10.3389/fimmu.2022.988479 (PMC9537444; doi:10.3389/fimmu.2022.988479)
Supplement: Supplementary file 2 [file Table_2.docx]

**Table S2** | TF-Gene topology table.

| **ID** | **Label** | **Degree** | **Betweenness** |
| --- | --- | --- | --- |
| 3434 | IFIT1 | 16 | 446.25 |
| 3437 | IFIT3 | 14 | 277.16 |
| 91543 | RSAD2 | 13 | 297.93 |
| 4939 | OAS2 | 13 | 276.79 |
| 4599 | MX1 | 12 | 306.5 |
| 282618 | IFNL1 | 11 | 258.99 |
| 2296 | FOXC1 | 10 | 359.72 |
| 2624 | GATA2 | 9 | 249.99 |
| 219285 | SAMD9L | 9 | 96.46 |
| 129607 | CMPK2 | 8 | 186.61 |
| 2537 | IFI6 | 8 | 105.05 |
| 2300 | FOXL1 | 7 | 127.64 |
| 3430 | IFI35 | 7 | 113.48 |
| 4938 | OAS1 | 7 | 111.05 |
| 5970 | RELA | 5 | 68.1 |
| 5468 | PPARG | 5 | 63.33 |
| 4782 | NFIC | 5 | 62.21 |
| 64135 | IFIH1 | 5 | 45.51 |
| 4940 | OAS3 | 5 | 34.54 |
| 3725 | JUN | 4 | 48.52 |
| 4205 | MEF2A | 4 | 48.43 |
| 4790 | NFKB1 | 4 | 47.92 |
| 6720 | SREBF1 | 4 | 42.44 |
| 3433 | IFIT2 | 4 | 38.39 |
| 25988 | HINFP | 4 | 33.29 |
| 4800 | NFYA | 4 | 32.21 |
| 6774 | STAT3 | 4 | 31.92 |
| 3429 | IFI27 | 4 | 19.3 |
| 7157 | TP53 | 3 | 38.1 |
| 672 | BRCA1 | 3 | 31.85 |
| 7392 | USF2 | 3 | 29.79 |
| 1385 | CREB1 | 3 | 25.06 |
| 8626 | TP63 | 3 | 23.49 |
| 5452 | POU2F2 | 3 | 22.88 |
| 2353 | FOS | 3 | 21.55 |
| 639 | PRDM1 | 3 | 14.03 |
| 7003 | TEAD1 | 3 | 9.97 |
| 5966 | REL | 2 | 7.59 |
| 6722 | SRF | 2 | 7.59 |
| 1051 | CEBPB | 2 | 6.24 |
| 6772 | STAT1 | 2 | 5.57 |
| 860 | RUNX2 | 2 | 5.39 |
| 7022 | TFAP2C | 2 | 4.99 |
| 2295 | FOXF2 | 2 | 4.99 |
| 7528 | YY1 | 2 | 4.68 |
| 7025 | NR2F1 | 2 | 4.03 |
| 2002 | ELK1 | 2 | 3.96 |
| 2908 | NR3C1 | 2 | 3.96 |
| 3727 | JUND | 2 | 2.58 |
| 2297 | FOXD1 | 1 | 0 |
| 2309 | FOXO3 | 1 | 0 |
| 4824 | NKX3-1 | 1 | 0 |
