# Supplementary material for: Bioinformatics and systems-biology analysis to determine the effects of Coronavirus disease 2019 on patients with allergic asthma
Source: Front Immunol. 2022 Sep 23;13:988479. doi: 10.3389/fimmu.2022.988479 (PMC9537444; doi:10.3389/fimmu.2022.988479)
Supplement: Supplementary file 3 [file Table_3.docx]

**Table S3** | MiRNA-Gene topology table.

| ID | Label | Degree | Betweenness | Expression |
| --- | --- | --- | --- | --- |
| 219285 | SAMD9L | 35 | 4677 | 0 |
| 3437 | IFIT3 | 27 | 2839.67 | 0 |
| 4939 | OAS2 | 19 | 2051 | 0 |
| 3434 | IFIT1 | 16 | 1879.17 | 0 |
| 4940 | OAS3 | 6 | 575 | 0 |
| MIMAT0000449 | hsa-mir-146a-5p | 5 | 4457.17 | 0 |
| 3433 | IFIT2 | 5 | 354.17 | 0 |
| 64135 | IFIH1 | 4 | 348 | 0 |
| 3429 | IFI27 | 3 | 887 | 0 |
| MIMAT0000416 | hsa-mir-1-3p | 3 | 531.17 | 0 |
| MIMAT0021123 | hsa-mir-5192 | 2 | 1960 | 0 |
| MIMAT0002873 | hsa-mir-502-5p | 2 | 672 | 0 |
| 91543 | RSAD2 | 2 | 565 | 0 |
| MIMAT0000083 | hsa-mir-26b-5p | 2 | 456 | 0 |
| MIMAT0000728 | hsa-mir-375 | 2 | 232 | 0 |
| MIMAT0000422 | hsa-mir-124-3p | 2 | 144.67 | 0 |
| 2537 | IFI6 | 2 | 117 | 0 |
| MIMAT0000765 | hsa-mir-335-5p | 2 | 117 | 0 |
| 4938 | OAS1 | 1 | 0 | 0 |
| MIMAT0000070 | hsa-mir-17-5p | 1 | 0 | 0 |
| MIMAT0000075 | hsa-mir-20a-5p | 1 | 0 | 0 |
| MIMAT0000093 | hsa-mir-93-5p | 1 | 0 | 0 |
| MIMAT0000097 | hsa-mir-99a-5p | 1 | 0 | 0 |
| MIMAT0000098 | hsa-mir-100-5p | 1 | 0 | 0 |
| MIMAT0000100 | hsa-mir-29b-3p | 1 | 0 | 0 |
| MIMAT0000252 | hsa-mir-7-5p | 1 | 0 | 0 |
| MIMAT0000264 | hsa-mir-203a-3p | 1 | 0 | 0 |
| MIMAT0000417 | hsa-mir-15b-5p | 1 | 0 | 0 |
| MIMAT0000426 | hsa-mir-132-3p | 1 | 0 | 0 |
| MIMAT0000435 | hsa-mir-143-3p | 1 | 0 | 0 |
| MIMAT0000444 | hsa-mir-126-5p | 1 | 0 | 0 |
| MIMAT0000455 | hsa-mir-185-5p | 1 | 0 | 0 |
| MIMAT0000680 | hsa-mir-106b-5p | 1 | 0 | 0 |
| MIMAT0000689 | hsa-mir-99b-5p | 1 | 0 | 0 |
| MIMAT0000735 | hsa-mir-380-3p | 1 | 0 | 0 |
| MIMAT0000754 | hsa-mir-337-3p | 1 | 0 | 0 |
| MIMAT0001341 | hsa-mir-424-5p | 1 | 0 | 0 |
| MIMAT0001413 | hsa-mir-20b-5p | 1 | 0 | 0 |
| MIMAT0002810 | hsa-mir-202-5p | 1 | 0 | 0 |
| MIMAT0002823 | hsa-mir-512-3p | 1 | 0 | 0 |
| MIMAT0002835 | hsa-mir-526b-5p | 1 | 0 | 0 |
| MIMAT0002851 | hsa-mir-517-5p | 1 | 0 | 0 |
| MIMAT0002853 | hsa-mir-519d-3p | 1 | 0 | 0 |
| MIMAT0003218 | hsa-mir-92b-3p | 1 | 0 | 0 |
| MIMAT0003235 | hsa-mir-570-3p | 1 | 0 | 0 |
| MIMAT0003238 | hsa-mir-573 | 1 | 0 | 0 |
| MIMAT0003243 | hsa-mir-578 | 1 | 0 | 0 |
| MIMAT0003289 | hsa-mir-620 | 1 | 0 | 0 |
| MIMAT0003315 | hsa-mir-645 | 1 | 0 | 0 |
| MIMAT0003320 | hsa-mir-650 | 1 | 0 | 0 |
| MIMAT0004549 | hsa-mir-148a-5p | 1 | 0 | 0 |
| MIMAT0004703 | hsa-mir-335-3p | 1 | 0 | 0 |
| MIMAT0004767 | hsa-mir-193b-5p | 1 | 0 | 0 |
| MIMAT0004795 | hsa-mir-574-5p | 1 | 0 | 0 |
| MIMAT0004982 | hsa-mir-939-5p | 1 | 0 | 0 |
| MIMAT0004985 | hsa-mir-942-5p | 1 | 0 | 0 |
| MIMAT0005573 | hsa-mir-1225-3p | 1 | 0 | 0 |
| MIMAT0005592 | hsa-mir-1237-3p | 1 | 0 | 0 |
| MIMAT0005900 | hsa-mir-1248 | 1 | 0 | 0 |
| MIMAT0005924 | hsa-mir-1270 | 1 | 0 | 0 |
| MIMAT0009979 | hsa-mir-2054 | 1 | 0 | 0 |
| MIMAT0010133 | hsa-mir-2110 | 1 | 0 | 0 |
| MIMAT0011157 | hsa-mir-2114-3p | 1 | 0 | 0 |
| MIMAT0015019 | hsa-mir-3147 | 1 | 0 | 0 |
| MIMAT0015023 | hsa-mir-3150a-3p | 1 | 0 | 0 |
| MIMAT0015052 | hsa-mir-3175 | 1 | 0 | 0 |
| MIMAT0016858 | hsa-mir-4306 | 1 | 0 | 0 |
| MIMAT0016886 | hsa-mir-4252 | 1 | 0 | 0 |
| MIMAT0017991 | hsa-mir-3613-3p | 1 | 0 | 0 |
| MIMAT0017995 | hsa-mir-3616-5p | 1 | 0 | 0 |
| MIMAT0018079 | hsa-mir-1273e | 1 | 0 | 0 |
| MIMAT0018095 | hsa-mir-3672 | 1 | 0 | 0 |
| MIMAT0018201 | hsa-mir-3926 | 1 | 0 | 0 |
| MIMAT0018940 | hsa-mir-4425 | 1 | 0 | 0 |
| MIMAT0018956 | hsa-mir-4438 | 1 | 0 | 0 |
| MIMAT0018977 | hsa-mir-4455 | 1 | 0 | 0 |
| MIMAT0019070 | hsa-mir-4531 | 1 | 0 | 0 |
| MIMAT0019692 | hsa-mir-4635 | 1 | 0 | 0 |
| MIMAT0019704 | hsa-mir-4644 | 1 | 0 | 0 |
| MIMAT0019751 | hsa-mir-4670-3p | 1 | 0 | 0 |
| MIMAT0019809 | hsa-mir-4708-5p | 1 | 0 | 0 |
| MIMAT0019853 | hsa-mir-4731-5p | 1 | 0 | 0 |
| MIMAT0019924 | hsa-mir-4770 | 1 | 0 | 0 |
| MIMAT0019969 | hsa-mir-4795-3p | 1 | 0 | 0 |
| MIMAT0020600 | hsa-mir-5095 | 1 | 0 | 0 |
| MIMAT0020601 | hsa-mir-1273f | 1 | 0 | 0 |
| MIMAT0021025 | hsa-mir-5003-5p | 1 | 0 | 0 |
| MIMAT0021081 | hsa-mir-5089-5p | 1 | 0 | 0 |
| MIMAT0022297 | hsa-mir-5589-5p | 1 | 0 | 0 |
| MIMAT0022717 | hsa-mir-873-3p | 1 | 0 | 0 |
| MIMAT0022742 | hsa-mir-1273g-3p | 1 | 0 | 0 |
| MIMAT0022970 | hsa-mir-3927-5p | 1 | 0 | 0 |
| MIMAT0023713 | hsa-mir-6088 | 1 | 0 | 0 |
| MIMAT0025460 | hsa-mir-6502-5p | 1 | 0 | 0 |
| MIMAT0025465 | hsa-mir-6504-3p | 1 | 0 | 0 |
| MIMAT0025468 | hsa-mir-6506-5p | 1 | 0 | 0 |
| MIMAT0025472 | hsa-mir-6508-5p | 1 | 0 | 0 |
| MIMAT0026622 | hsa-mir-619-5p | 1 | 0 | 0 |
| MIMAT0026639 | hsa-mir-1301-5p | 1 | 0 | 0 |
| MIMAT0026740 | hsa-mir-1250-3p | 1 | 0 | 0 |
| MIMAT0027038 | hsa-mir-1343-5p | 1 | 0 | 0 |
| MIMAT0027363 | hsa-mir-6731-5p | 1 | 0 | 0 |
| MIMAT0027403 | hsa-mir-6751-3p | 1 | 0 | 0 |
| MIMAT0027426 | hsa-mir-6763-5p | 1 | 0 | 0 |
| MIMAT0027493 | hsa-mir-6796-3p | 1 | 0 | 0 |
| MIMAT0027514 | hsa-mir-6807-5p | 1 | 0 | 0 |
| MIMAT0027520 | hsa-mir-6810-5p | 1 | 0 | 0 |
| MIMAT0027547 | hsa-mir-6823-3p | 1 | 0 | 0 |
| MIMAT0027550 | hsa-mir-6825-5p | 1 | 0 | 0 |
| MIMAT0027614 | hsa-mir-6857-5p | 1 | 0 | 0 |
| MIMAT0027629 | hsa-mir-6864-3p | 1 | 0 | 0 |
| MIMAT0027634 | hsa-mir-6867-5p | 1 | 0 | 0 |
| MIMAT0027637 | hsa-mir-6868-3p | 1 | 0 | 0 |
| MIMAT0028213 | hsa-mir-7151-3p | 1 | 0 | 0 |
| MIMAT0030418 | hsa-mir-6516-3p | 1 | 0 | 0 |
| MIMAT0030994 | hsa-mir-8067 | 1 | 0 | 0 |
| MIMAT0031012 | hsa-mir-8085 | 1 | 0 | 0 |
| MIMAT0031074 | hsa-mir-450a-2-3p | 1 | 0 | 0 |
| MIMAT0031119 | hsa-mir-1199-5p | 1 | 0 | 0 |
