# Supplementary material for: Bioinformatics and systems-biology analysis to determine the effects of Coronavirus disease 2019 on patients with allergic asthma
Source: Front Immunol. 2022 Sep 23;13:988479. doi: 10.3389/fimmu.2022.988479 (PMC9537444; doi:10.3389/fimmu.2022.988479)
Supplement: Supplementary file 4 [file DataSheet_1.docx]

**Supplementary Materials**


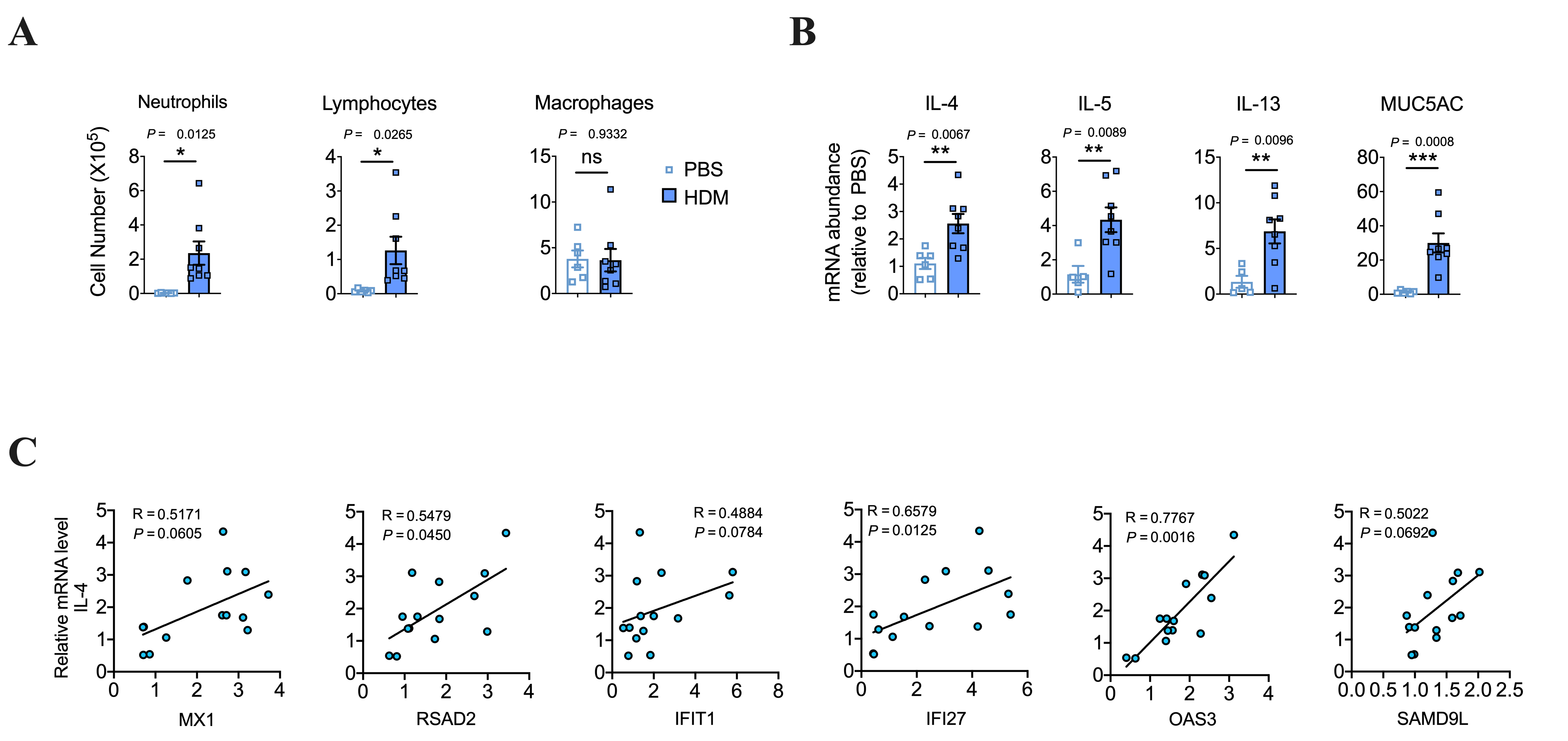


**Supplementary Figure 1 |** Validation of the identified hub genes with a murine model of asthma. **(A)** Differential cell counts in BALF samples from PBS- or HDM-treated mice. The data shown were combined from two experiments. *n* = 6–8. **(B)** Cytokine mRNA abundances in homogenized lung tissues. The data shown were combined from two experiments. *n* = 6–8. (**C**) Corresponding scatterplot showing the relationships between mRNA-expression levels of the identified hub genes and IL-4 in the lungs, as determined by Spearman’s rank correlation (R). Student’s *t*-test was used to evaluate the differences. **P* < 0.05; ***P* < 0.01; ****P* < 0.001. The results shown are presented as the mean ± SEM.
